# Alterations of the Ca^2+^ clearing mechanisms by type 2 diabetes in aortic smooth muscle cells of Zucker diabetic fatty rat

**DOI:** 10.3389/fphys.2023.1200115

**Published:** 2023-05-11

**Authors:** Adriana Moreno-Salgado, Nayeli Coyotl-Santiago, Roberto Moreno-Vazquez, Mayte Lopez-Teyssier, Mario Garcia-Carrasco, Francesco Moccia, Roberto Berra-Romani

**Affiliations:** ^1^ Department of Biomedicine, School of Medicine, Benemerita Universidad Autonoma de Puebla, Puebla, Mexico; ^2^ Department of Immunology, School of Medicine, Benemerita Universidad Autonoma de Puebla, Puebla, Mexico; ^3^ Department of Biology and Biotechnology “Lazzaro Spallanzani”, University of Pavia, Pavia, Italy

**Keywords:** type 2 diabetes mellitus, intracellular Ca^2+^, Fura-2, freshly isolated vascular smooth muscle cells, Zucker diabetic fatty

## Abstract

Type 2 Diabetes Mellitus (T2DM) is a rapidly rising disease with cardiovascular complications constituting the most common cause of death among diabetic patients. Chronic hyperglycemia can induce vascular dysfunction through damage of the components of the vascular wall, such as vascular smooth muscle cells (VSMCs), which regulate vascular tone and contribute to vascular repair and remodeling. These functions are dependent on intracellular Ca^2+^ changes. The mechanisms by which T2DM affects Ca^2+^ handling in VSMCs still remain poorly understood. Therefore, the objective of this study was to determine whether and how T2DM affects Ca^2+^ homeostasis in VSMCs. We evaluated intracellular Ca^2+^ signaling in VSMCs from Zucker Diabetic Fatty rats using Ca^2+^ imaging with Fura-2/AM. Our results indicate that T2DM decreases Ca^2+^ release from the sarcoplasmic reticulum (SR) and increases the activity of store-operated channels (SOCs). Moreover, we were able to identify an enhancement of the activity of the main Ca^2+^ extrusion mechanisms (SERCA, PMCA and NCX) during the early stage of the decay of the ATP-induced Ca^2+^ transient. In addition, we found an increase in Ca^2+^ entry through the reverse mode of NCX and a decrease in SERCA and PMCA activity during the late stage of the signal decay. These effects were appreciated as a shortening of ATP-induced Ca^2+^ transient during the early stage of the decay, as well as an increase in the amplitude of the following plateau. Enhanced cytosolic Ca^2+^ activity in VSMCs could contribute to vascular dysfunction associated with T2DM.

## 1 Introduction

Diabetes mellitus (DM) comprises a group of metabolic diseases characterized by an increase in plasma glucose levels (hyperglycemia) due to defects in the secretion (type 1 DM or T1DM) or action (type 2 DM or T2DM) of insulin (ElSayed et al., 2023). T2DM is the most common form of DM as it constitutes 90% of all diabetic cases worldwide (IDF Diabetes Atlas, 2021). The most common cause of death among diabetic patients is represented by cardiovascular disorders with heart disease causing approximately 70% of patients’ deaths (Gu et al., 1998; Einarson et al., 2018). Other causes of mortality include cerebrovascular disease, myocardial infarction, hypertension and atherosclerosis (Kennedy, 2017).

It has long been known that hyperglycemia, dyslipidemia and insulin resistance, all of which accompany T2DM, are related to an increase in cardiovascular risk by affecting the structural components of blood vessels through multiple mechanisms, such as protein glycosylation and oxidative stress, which lead to vascular dysfunction (Szuszkiewicz-Garcia and Davidson, 2014; Mao et al., 2022). Vascular dysfunction is a complex and multifactorial process; however, there is strong evidence showing that T2DM alters intracellular Ca^2+^ handling in vascular smooth muscle cells (VSMCs) (Fernandez-Velasco et al., 2014; Nieves-Cintron et al., 2021). Remodeling of Ca^2+^ handling in VSMCs may dramatically alter vascular reactivity as well other processes, such as migration and proliferation, which are involved in vascular repair and angiogenesis (Fernandez-Velasco et al., 2014). Alterations in these processes may favor the development of pathologies such as hypertension and atherosclerosis. Although most of the studies performed in animal models of T2DM hint at an increase in the bioavailability of intracellular Ca^2+^ that enhances vascular reactivity or cell migration and proliferation, depending on the VSMC phenotype (Fernandez-Velasco et al., 2014), the cellular and molecular mechanisms involved still remain poorly understood. Several reports suggest that T2DM induces an increase in the expression or activity of Ca^2+^-permeable channels, such as the *store-operated calcium entry* (SOCE) pathway and *transient receptor potential canonical* channels (TRPC) on the sarcolemma (Chung et al., 2009; Nieves-Cintron et al., 2021), and *inositol 1,4,5-trisphosphate receptor* (IP_3_R) Ca^2+^ release channels in the sarcoplasmic reticulum (SR) (Velmurugan and White, 2012; Nieves-Cintron et al., 2021). For instance, a recent study suggested that SOCE is enhanced in diabetic VSMCs and exacerbates vasoconstriction in the aorta of Zucker Diabetic Fatty (ZDF) rats, a model widely used for the study of T2DM (Yang et al., 2020). In addition, hyperglycemia stimulates expression of ORAI1, thus, augmenting store-operated Ca^2+^-entry in primary human aortic smooth muscle cells (Ma et al., 2020).

Only scarce information is available about the alteration of Ca^2+^ removal mechanisms, which significantly contribute to maintain intracellular Ca^2+^ homeostasis under physiological conditions, in VSMCs during T2DM. A study carried out on cultured A7r5 cells showed that hyperglycemia causes an increase in the activity of Plasma Membrane Ca^2+^-ATPase (PMCA), which extrudes Ca^2+^ across the sarcolemma, as well as a decrease in the activity of Sarco-Endoplasmic Reticulum Ca^2+^-ATPase (SERCA), which sequesters cytosolic Ca^2+^ into the SR (El-Najjar et al., 2017). Similarly, it has been suggested that SERCA activity is downregulated by oxidative stress in rat aortic VSMCs from ZDF rats, although the impact of SERCA oxidation of Ca^2+^ clearance has not been directly assessed (Tong et al., 2010). Na^+^/Ca^2+^ exchanger (NCX) represents an alternative pathway for Ca^2+^ extrusion across the plasma membrane in VSMCs, but it is still unknown whether it is affected by T2DM. Therefore, it is mandatory to gain further insights about the remodeling of the Ca^2+^ handling machinery in an experimental model that effectively recapitulates the pathophysiology of T2DM, such as ZDF rats. The present investigation sought to exhaustively assess whether and how T2DM alters the intracellular Ca^2+^ signaling in VSMCs from the aorta of ZDF rats, loaded with the Ca^2+^-sensitive indicator, Fura-2-acetoxymethyl ester (Fura-2/AM).

## 2 Materials and methods

### 2.1 Zucker diabetic fatty rat model

All the experiments were carried out according to the *Norma Oficial Mexicana* (NOM-062-ZOO-1999, 9.4.2.1.3.) for the care and handling of laboratory animals. The protocols were reviewed and approved by the Animal Care and Use Committee of the Benemerita Universidad Autonoma de Puebla, identification code: BERRSAL71, 18-05-2017. Experiments were carried out in male ZDF rats (3 months old) from Charles River Laboratories, California, U.S.A. Throughout the text, diabetic-obese ZDF rats (ZDF-Lepr^fa/fa^) will be designated as OZDF rats, and lean controls, non-obese non-diabetic ZDF (ZDF-Lepr^+/+^) as LZDF. The rats were kept at the University Animal Core Facilities under controlled environmental temperature and, exposed to light-dark cycles of 12 h, with *ad libitum* consumption of water and high energy diet, Purina 5,008 chow.

### 2.2 Somatic and biochemical parameters

To determine if OZDF rats did indeed develop T2DM, 5 days prior to the experiment, a glucose tolerance test was performed. The animals were fasted for 6 h in metabolic cages with free access to water. Immediately after, a glucose dose (2 g/kg of weight) was administrated intraperitoneally. Two hours later, a blood sample was obtained by puncture of the caudal vein to measure glucose levels using the Accucheck^®^ system (Roche, Mannheim, Germany). The day of the experiment, we proceeded to measure the body mass (weight), length (distance from the tip of the nose to the base of the tail) and the abdominal circumference using a measuring tape.

The body mass index (BMI) was calculated using the following equation:

$$BMI=\frac{Body\;mass\;\left(g\right)}{{Length}^{2}\;{\left(cm\right)}^{2}}$$

Finally, all the epididymal fat surrounding both testes was accurately removed and weighed.

### 2.3 Isolation and culture rat aortic VSMC and dissection of epididymal fat samples

Isolation and culture of rat VSMCs were carried out according to the protocol previously described by Berra-Romani et al. to isolate proliferating, non-contracting VSMCs (Berra-Romani et al., 2008). ZDF rats were anesthetized with intraperitoneal ketamine-xylazine solution, 0.2 mL per 100 g of weight. The thoracic aorta was dissected out and perfused with low-Ca^2+^ physiological salt solution (PSS1, see composition in Section 2.5). Under sterile conditions the artery was cleaned of fat and connective tissue and then incubated for ∼40 min at 37°C in PSS1 containing 1 mg/mL collagenase type 2. After the incubation, the aorta was washed three times with PSS1 and the adventitia was carefully stripped. The aorta was cut longitudinally in order to expose the *tunica intima* and the endothelium was mechanically removed by rubbing a microdissection scissor against the endothelial layer. Aortic tissue was cut in a proximately 2 mm pieces and enzymatically digested for 40 min at 37°C in PSS1 containing 2 mg/mL collagenase type XI, 0.16 mg/mL elastase type IV and 2 mg/mL bovine serum albumin (BSA; fat free). After de digestion, VSMCs were resuspended by gently triturating the tissue with a fire-polished Pasteur pipette. To stop enzymatic digestion, 10 mL of Dulbecco’s modified Eagle’s medium (DMEM) supplemented with 10% fetal bovine serum (FBS) was added. VSMCs were centrifugated for 10 min at 300*g*. The supernatant was discarded, and the resulting cell pellet was re-suspended in 1 mL complete medium (DMEM with 10% FBS and 1% antibiotic antimycotic solution (100x)). VSMC were plated on 35 mm culture dishes under a humidified atmosphere of 5% CO_2_ - 95% O_2_ at 37°C. Culture medium was changed on days 4 and 7 and Ca^2+^ imaging experiments were performed on subconfluent cultures on days 7 and 8. The aortic VSMCs identity was confirmed by α-actin labelling, as shown in (Berra-Romani et al., 2008).

### 2.4 [Ca^2+^]_i_ measurements

Cultured VSMCs were loaded with 3 µM Ca^2+^ indicator, Fura-2/AM in PSS2 (see composition in Section 2.5) for 35 min at controlled room temperature (22–23 °C). After 20 min washing in PSS2, the culture dish was mounted onto the stage of an upright epifluorescence Axiolab microscope (Carl Zeiss, Oberkochen, Germany) equipped with a ×40 Achroplan objective (water-immersion, 2.05 mm working distance, 1.0 numerical aperture) to observe the cells. VSMCs were excited alternately at 340 and 380 nm, and the emitted light was detected at 510 nm. A neutral density filter (optical density = 1.0) was coupled to the 380 nm filter to approach the intensity of the 340 nm light. A round diaphragm was used to increase the contrast. The exciting filters were mounted on a filter wheel equipped with a shutter (Lambda 10, Sutter Instrument, Novato, CA, United States). Custom software, working in the LINUX environment, was used to drive the camera (Extended-ISIS Camera, Photonic Science, Millham, United Kingdom) and the filter wheel, and to measure and plot on-line the fluorescence from a number of 20–50 rectangular “regions of interest” (ROI) enclosing 20–50 single cells. Each ROI was identified by a number. The intracellular Ca^2+^ concentration ([Ca^2+^]_i_) was monitored by measuring, for each ROI, the ratio of the mean fluorescence emitted at 510 nm when exciting alternatively at 340 and 380 nm (shortly termed “Ratio (F_340_/F_380_)”. An increase in [Ca^2+^]_i_ causes an increase in the Ratio (F_340_/F_380_). Ratio measurements were performed and plotted on-line every 3 s. Ratio (F_340_/F_380_) values are expressed as arbitrary units (a.u.). Images were stored on the hard disk and converted offline to Ratio (F_340_/F_380_) images by ImageJ software (National Institutes of Health, United States, http://rsbweb.nih.gov/ij/). The experiments were carried out at controlled room temperature (22–23°C) to limit time-dependent decreases in the intensity of the fluorescence signal.

Mn^2+^ has been shown to quench Fura-2 fluorescence. Since Mn^2+^ and Ca^2+^ share common entry pathways in the plasma membrane, Fura-2 quenching by Mn^2+^ is regarded as an index of divalent cation influx. Experiments were carried out at the 360 nm wavelength, the isosbestic wavelength for Fura-2, and in Ca^2+^-free medium supplemented with 0.5 mM EGTA, as previously described (Zuccolo et al., 2019). This avoids Ca^2+^ competition for Mn^2+^ entry and, therefore, enhances Mn^2+^ quenching.

### 2.5 Solutions

Low-Ca^2+^ physiological salt solution (PSS1) had the following composition (in mM): 140 NaCl, 5.36 KCl, 0.34 Na_2_HPO_4_, 0.44 KH_2_PO_4_, 10 HEPES, 1.2 MgCl_2_, 10 D-glucose and 0.05 CaCl_2_. Physiological salt solution (PSS2) composition (in mM) was: 140 NaCl, 5 KCl, 1.2 NaHPO_4_, 5 NaHCO_3_, 10 HEPES, 1.4 MgCl_2_, 1.8 CaCl_2_ and 11.5 D-glucose. In Ca^2+^ free solution (0Ca^2+^), 0.05 EGTA was added. In Mn^2+^-quenching experiments, 200 µM MnCl_2_ was added to the 0Ca^2+^ external solution. Solutions were titrated to pH 7.2 for PSS1 and 7.4 for PSS2 with NaOH. The osmolality of PSS as measured with an osmometer (Wescor 5,500, Logan, UT) was 338 mmol/kg.

### 2.6 Data analysis

For each protocol, data were collected from at least four rats under each condition. The amplitude of the peak Ca^2+^ response to either cyclopiazonic acid (CPA) or adenosine-trisphosphate (ATP) was measured as the difference between the Ratio (F_340_/F_380_) at the peak and the mean Ratio (F_340_/F_380_) of 500 s baseline before the peak (Supplementary Figure S1). Amplitude of the late stage of the decay (Amp600) was calculated as the difference between the Ratio (F_340_/F_380_) 600 s after adding the agonist and the mean Ratio (F_340_/F_380_) of 200 s baseline before the peak of the Ca^2+^ response (Supplementary Figure S1). The duration of the Ca^2+^ response to ATP was measured as the time it takes the Ca^2+^ signal to be reduced at 90% (early) (Supplementary Figure S1), 60% (intermediate) (Supplementary Figure S1) and 30% (late) (Supplementary Figure S1) of the initial Ca^2+^ peak amplitude (considered as 100%), shorty termed as “decay time” (DT) (Supplementary Figure S1). The area under the curve (AUC) was measured by calculating the integral of each Ca^2+^ tracing from when the ATP is applied until it is removed. In order to perform the statistical comparison between the experimental groups in the presence of selective inhibitors of Ca^2+^- clearing mechanisms, we proceeded to normalize the measures to the mean value of each parameter determined in the absence of the inhibitor within the same experimental group (shortly termed “Δ”). For mean traces, fluorescence levels (F) were normalized to resting fluorescence (F_0_) to compare the height of the Ca^2+^ responses produced by cells displaying different basal fluorescence levels (F/F_0_). The rate of Mn^2+^ influx was evaluated by measuring the slope of the fluorescence intensity curve after Mn^2+^ addition (Zuccolo et al., 2019). Data are expressed as mean ± standard error (SE). Normal data (identified using the D’Agostino and Pearson omnibus normality test (*p* < 0.05)), were statistically analyzed using an unpaired Student’s t-test. A *p*-value < 0.05 was considered statistically significant.

### 2.7 Chemicals

SEA0400 was obtained from Tocris Bioscience (Bristol, United Kindom). Collagenase type 2 and FBS were obtained from Gibco (GIBCO BRL, Life Technologies, Grand Island, NY). All other chemicals were purchased from Sigma-Aldrich.

## 3 Results

### 3.1 Zucker diabetic fatty rat characteristics

Somatic parameter obtained from 3 months old littermate LZDF and OZDF rats are shown in Table 1. OZDF rats presented an increase of around 66% in body mass weight (*p* ≤ 0.05) and of around 6.5% of the nose-tail length (*p* ≤ 0.05) compared with their control, LZDF rats. Given that the increase in weight could be attributed to an increase in the rat size (to its length), we proceeded to calculate the body mass index (BMI) to rule out that the differences seen in the weight were due to the difference in length between both groups. Likewise, the mean BMI value was statistically higher (46.6%) in the OZDF rats compared to the LZDF group (*p* ≤ 0.05), suggesting that the weight gain is due to obesity that has been widely reported in the literature in this strain of rats (Shiota and Printz, 2012; Wang et al., 2013; Al-Awar et al., 2016; King and Bowe, 2016; Berra-Romani et al., 2019). The presence of obesity in the OZDF rats was demonstrated by the 28% and 405% increase in abdominal circumference (*p* ≤ 0.05) and epididymal fat weight (*p* ≤ 0.05), respectively, in OZDF vs. LZDF rats. To confirm T2DM presence in OZDF rats, a glucose tolerance test was performed as described in Materials and Methods section. The mean ± SE of blood glucose levels measured 2 h after the intraperitoneal administration of glucose (2 g/kg of weight), was approximately 90% higher (*p* ≤ 0.05) in the OZDF rats compared to the LZDF rats (Table 1). Altogether the results confirm that OZDF rats present obesity and diabetes. In addition, a deeper biochemical analysis of LZDF rats and OZDF rats has been presented in (Berra-Romani et al., 2019), showing that OZDF rats present: 1) hyperlipidemia (increased total cholesterol, very low-density lipoproteins, and triglyceride levels); 2) hyperinsulinemia both in fasting and glucose-fed animals; and 3) insulin resistance. All together these results confirm the presence in OZDF rats of the main characteristics of human T2DM.

**TABLE 1:** Zucker Diabetic Fatty rat characteristics. Mean ± SE of somatic and biochemical parameters measured in Zucker Diabetic Fatty (LZDF and OZDF) rats at the age of 12 weeks. Body mass index (BMI) was calculated according to the equation described in materials and Methods. Blood glucose levels were measured using the Accucheck^®^ system, 2 h after the intraperitoneal administration of glucose (2 g/kg of weight) in 6 h fasted animals. * indicates *p* ≤ 0.05 (Student’s t-test, n = 5 per group; for glucose values n = 14 per group).

	LZDF	OZDF
Body weight (g)	297.8 ± 3.87	494.3 ± 5.86*
Length (cm)	22.19 ± 0.25	23.64 ± 0.36*
BMI (g/cm2)	0.60 ± 0.01	0.88 ± 0.02*
Abdominal circumference (cm)	13.35 ± 0.15	17.13 ± 0.27*
Epididymal fat (g)	2.50 ± 0.15	12.63 ± 0.72*
Glucose 2h (gr/dl)	126.06 ± 5.49	239.5 ± 24.54*

### 3.2 T2DM shortens the early phase of the decay and increases the plateau amplitude of ATP-induced Ca^2+^ signals in rat aortic VSMCs

**FIGURE 1:**
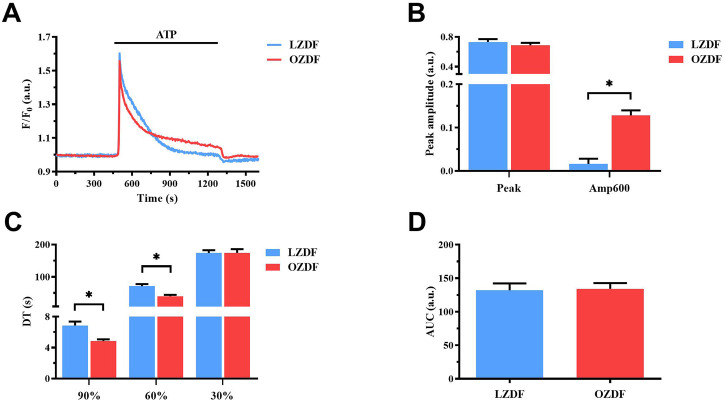
T2DM shortens the ATP-induced Ca^2+^ signal during the early phase of the decay and increases the plateau amplitude in rat aortic VSMCs. Mean traces of the Ca^2+^ signal evoked by ATP (300 μM) in VSMCs from LZDF (blue tracing) and OZDF (red tracing) rats **(A)**. Peak amplitude [**(B)**, left)], amplitude 600 s after the application of the agonist (Amp600) [**(B)**, right)], decay time at 90, 60% and 30% of total amplitude (DT) **(C)** and area under the curve (AUC) **(D)** are expressed as mean ± SE. Statistical comparison between groups was performed using Student’s t-test. * indicates *p* < 0.05. Analysis was performed in 121 cells obtained from 6 rats for the LZDF group (*n* = 6; 121 cells) and 179 cells obtained from 6 rats for the OZDF group (*n* = 6; 179 cells).

Preliminary recordings revealed that resting Ca^2+^ levels were similar (Supplementary Figure S2A) and that spontaneous Ca^2+^ oscillations (Supplementary Figure S2B) did not occur in VSMCs from the two animal groups. Moreover, 20 µM ATP was found to induce only a Ca^2+^ transient in rat aortic VSMCs from both OZDF and LZDF rats (Supplementary Figure S3). Since we were also interested in the impact of T2DM on the plateau phase of the Ca^2+^ response to physiological autacoids (Berra-Romani et al., 2019), ATP concentration was raised to 300 µM. In resting VSMCs from both LZDF and OZDF rats, the application of 300 µM ATP induced an intracellular Ca^2+^ signal that consisted in a rapid Ca^2+^ spike followed by a slow decay phase to a sustained plateau phase, which returned to the baseline upon agonist removal (Figure 1A). When comparing both groups, we did not find any statistical difference in both the peak amplitude (Figure 1B, left panel) and area under the curve (AUC) (Figure 1D). However, decay time (DT) at 90% and 60% of total amplitude were decreased (*p* ≤ 0.05) in VSMCs from OZDF rats (Figure 1C). In addition, we found an increase in the amplitude of the plateau phase in VSMCs from OZDF rats compared to LZDF rats, as shown by the increase in Amp600 value (*p* ≤ 0.05) (Figure 1B, right panel). Conversely, the decay time at 30% was not different between both groups (Figure 1C). The fastest decline of the initial Ca^2+^ peak could explain why the increase in Amp600 does not result in an increased AUC.

### 3.3 Effect of the removal of extracellular Ca^2+^ (0Ca^2+^) on ATP-induced Ca^2+^ signals in rat aortic VSMCs

**FIGURE 2:**
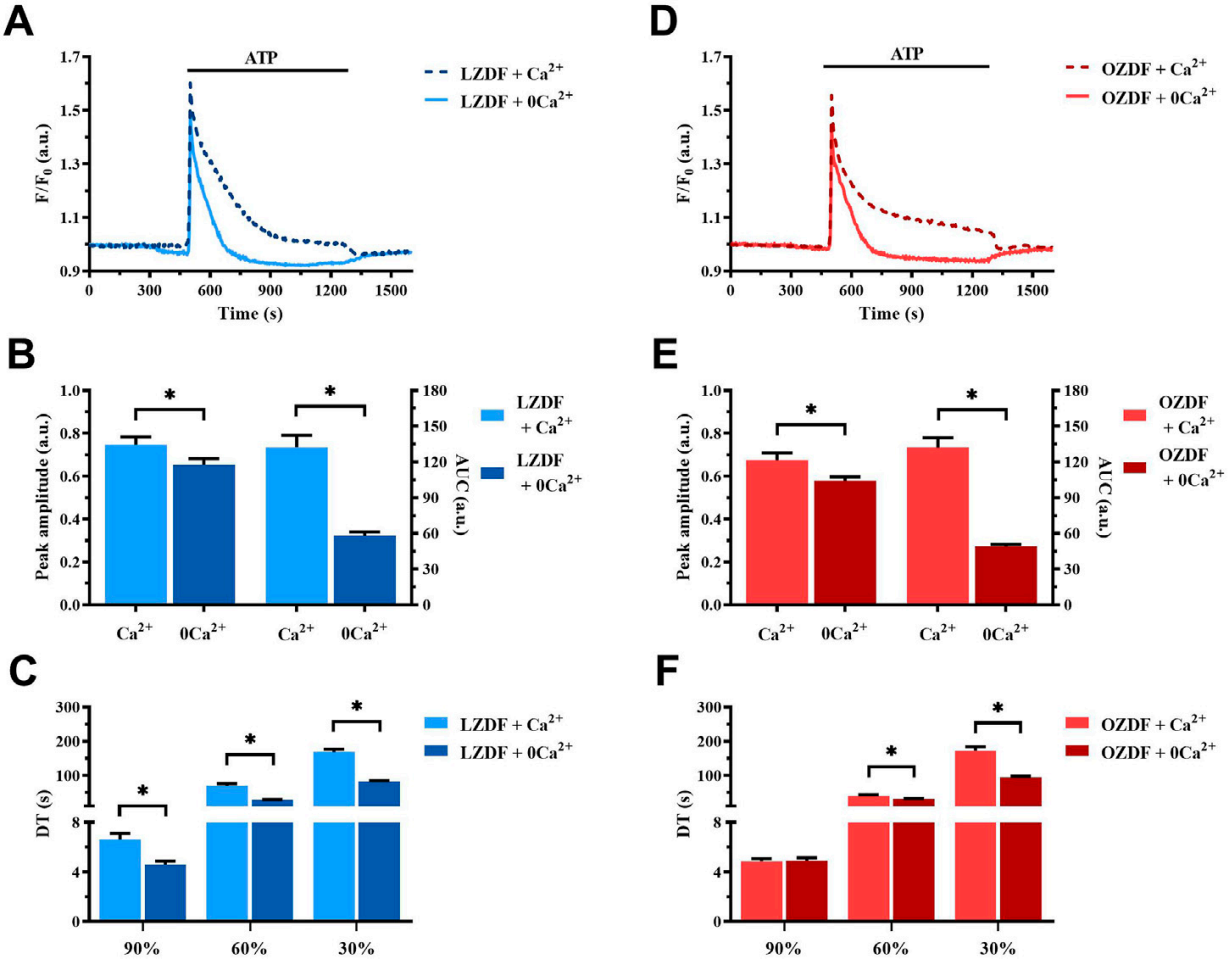
Effect of the removal of extracellular Ca^2+^ (0Ca^2+^) on the ATP-induced Ca^2+^ signal in rat aortic VSMCs. Mean traces of the Ca^2+^ signal evoked by ATP (300 μM) in the absence (continuous light-blue line; 0Ca^2+^) and presence (dashed dark-blue line; Ca^2+^) of extracellular Ca^2+^ in VSMCs from LZDF rats **(A)**. Peak amplitude [**(B)**, left)], area under the curve (AUC) [**(B)**, right)] and decay time (DT) **(C)** of the Ca^2+^ signal in VSMCs from LZDF rats, in presence (light blue bars; Ca^2+^) and absence (dark blue bars; 0Ca^2+^). Mean traces of the Ca^2+^ signal evoked by ATP (300 μM) in the absence (continuous light-red line; 0Ca^2+^) and presence (dashed dark-red line; Ca^2+^) of extracellular Ca^2+^ in VSMCs from OZDF rats **(D)**. Peak amplitude [**(E)**, left)], AUC [**(E)**, right)] and DT **(F)** of the Ca^2+^ signal in VSMCs from OZDF rats. All parameters are expressed as mean ± SE. Statistical comparison between groups was performed using Student’s t-test. * indicates *p* < 0.05 (*n* = 6; 127 cells for LZDF Ca^2+^, *n* = 6; 191 cells for LZDF 0Ca^2+^, *n* = 6; 182 cells for OZDF Ca^2+^, *n* = 6; 204 cells for OZDF 0Ca^2+^).

Figure 2 shows the average of Ca^2+^ signals recorded from cultured VSMCs obtained from LZDF (Figure 2A) and OZDF (Figure 2D) rat aortas challenged with ATP (300 μM) in presence (dotted line; + Ca^2+^) and absence (continuous line; + 0Ca^2+^) of Ca^2+^ in the extracellular medium. Removal of extracellular Ca^2+^ (0Ca^2+^) caused a decrease in peak amplitude, AUC, and decay time, as well as the disappearance of the plateau phase, in ATP-induced Ca^2+^ transients in both LZDF (Figures 2A-C) and OZDF VSMCs (Figures 2D–F). These findings confirm that both endogenous Ca^2+^ release through ER-resident IP_3_Rs and extracellular Ca^2+^ entry via store-operated channels (SOCs) support the Ca^2+^ response to ATP in proliferating rat aortic VSMCs (Berra-Romani et al., 2008). In accord, depletion of the ER Ca^2+^ store with cyclopiazonic acid (CPA; 10 µM) prevented the subsequent Ca^2+^ response to ATP (300 µM) (Supplementary Figure S4). Of note, caffeine (10 mM), a selective agonist of ryanodine receptors (RyR) (Pulina et al., 2010) failed to elicit an increase in [Ca^2+^]_i_ in rat aortic VSMCs from LZDF rats (Supplementary Figure S3), which is consistent with the notion that proliferating VSMCs lack RyRs (Berra-Romani et al., 2008). Therefore, IP_3_Rs drive ATP-dependent SR Ca^2+^ mobilization under 0Ca^2+^ conditions. Furthermore, the Mn^2+^-quenching assay, which has been widely employed to measure SOCE in both vascular endothelial cells (Zuccolo et al., 2019) and VSMCs (Smani et al., 2007), showed that CPA caused an increase in the rate of Fura-2 fluorescence quenching by extracellular Mn^2+^ (Supplementary Figure S5). However, the rate of CPA-evoked Mn^2+^ entry was significantly (*p* < 0.05) enhanced in rat aortic VSMCs from OZDF rats (Supplementary Figure S5), thereby confirming SOCE upregulation by T2DM.

### 3.4 T2DM reduces agonist-induced SR Ca^2+^ release in rat aortic VSMCs

**FIGURE 3:**
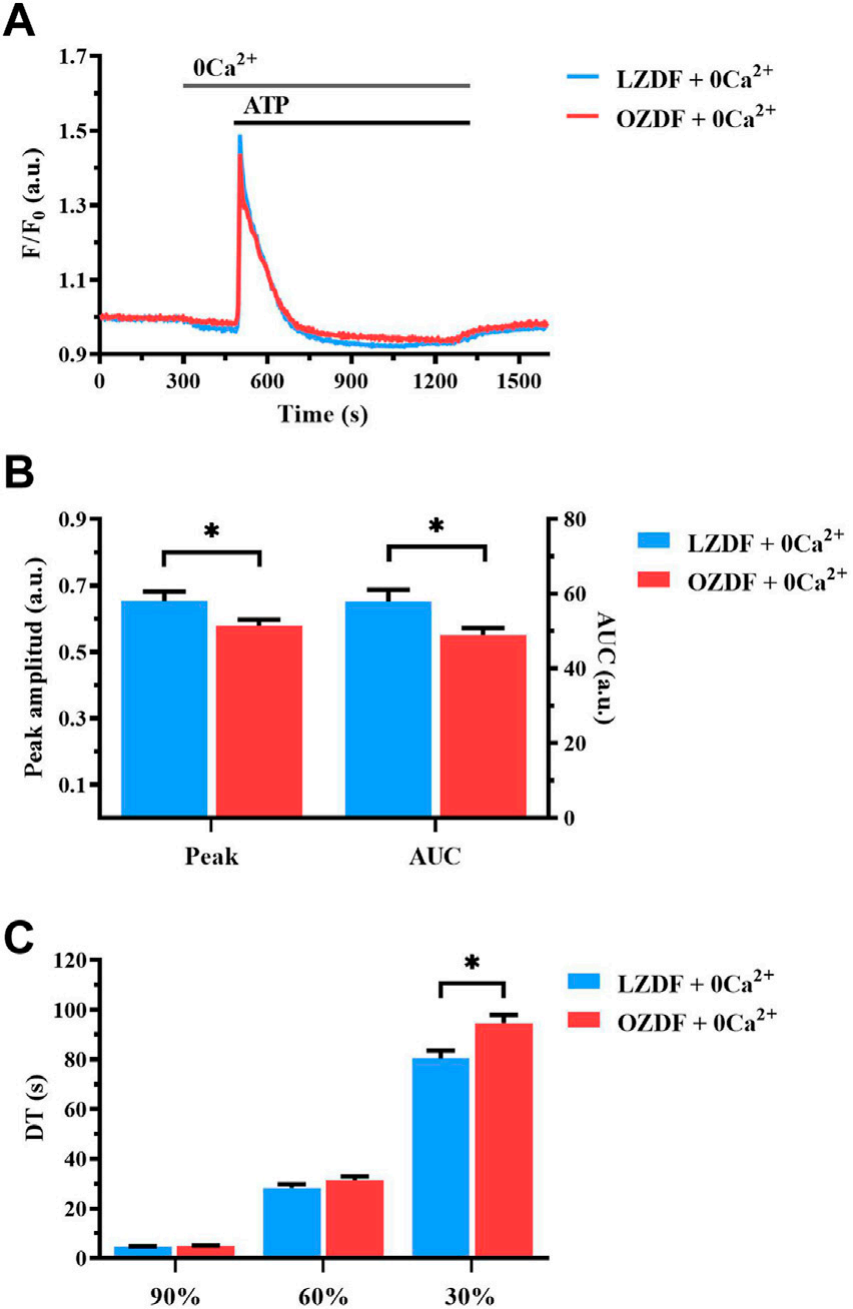
T2DM reduces agonist-induced Ca^2+^ release from SR in rat aortic VSMCs. Mean traces of the Ca^2+^ signal evoked by ATP (300 μM) in the absence of extracellular Ca^2+^ (0Ca^2+^) in VSMCs from LZDF (blue tracing) and OZDF (red tracing) rats **(A)**. Peak amplitude [**(B)**, left)], area under the curve (AUC) [**(B)**, right)] and DT **(C)** of the Ca^2+^ signal in VSMCs from LZDF (blue bars) and OZDF (red bars) rats. All parameters are expressed as mean ± SE. Statistical comparison between groups was performed using Student’s t-test. * indicates *p* < 0.05 (n = 6; 191 cells for LZDF 0Ca^2+^, *n* = 6; 204 cells for OZDF 0Ca^2+^).

In order to assess whether IP_3_-induced SR Ca^2+^ release is affected by T2DM, we compared intracellular Ca^2+^ release evoked by the IP_3_-synthesizing autacoid, ATP (300 µM), in the absence of extracellular Ca^2+^ (0Ca^2+^) in rat aortic VSMCs from LZDF and OZDF rats (Figure 3A) (Berra-Romani et al., 2008; Berra-Romani et al., 2019). Under these conditions, we found a reduction in both the peak Ca^2+^ amplitude and AUC of the Ca^2+^ transient in OZDF as compared to LZDF VSMCs (Figure 3B). Of note, the peak Ca^2+^ response to ATP was lower in rat aortic OZDF VSMCs in the absence, but not in the presence, of extracellular Ca^2+^ (see Figure 1B). We did not find any statistical difference in the decay time to 90% and 60% of the total amplitude; however, the decay time to 30% of the Ca^2+^ response was significantly (*p* < 0.05) increased in VSMCs from OZDF rats (Figure 3C). Conversely, in the presence of extracellular Ca^2+^, the early phase of the decay was faster in OZDF VSMCs and there was no difference in the decay time at 30% between the two animal groups (see Figure 1C). These observations suggest that: 1) endogenous Ca^2+^ release is reduced and/or 2) the Ca^2+^-transporting mechanisms clearing the initial Ca^2+^ peak are altered by T2DM, although this dysregulation is unmasked by removing extracellular Ca^2+^. The fastest decline of the initial Ca^2+^ peak recorded in the presence of external Ca^2+^ (Figure 1C) suggests that extracellular Ca^2+^ entry could stimulate SERCA, PMCA and/or NCX, as previously demonstrated in rat aortic VSMCs (Baryshnikov et al., 2009), and thereby accelerate cytosolic Ca^2+^ clearance (see Discussion). In addition, enhanced SOCE is also likely to compensate for the lower IP_3_-induced SR Ca^2+^ release and bring the amplitude of the initial Ca^2+^ peak to the same value as that measured in LZDF rats.

### 3.5 T2DM does not alter SR Ca^2+^ content in rat aortic VSMCs

**FIGURE 4:**
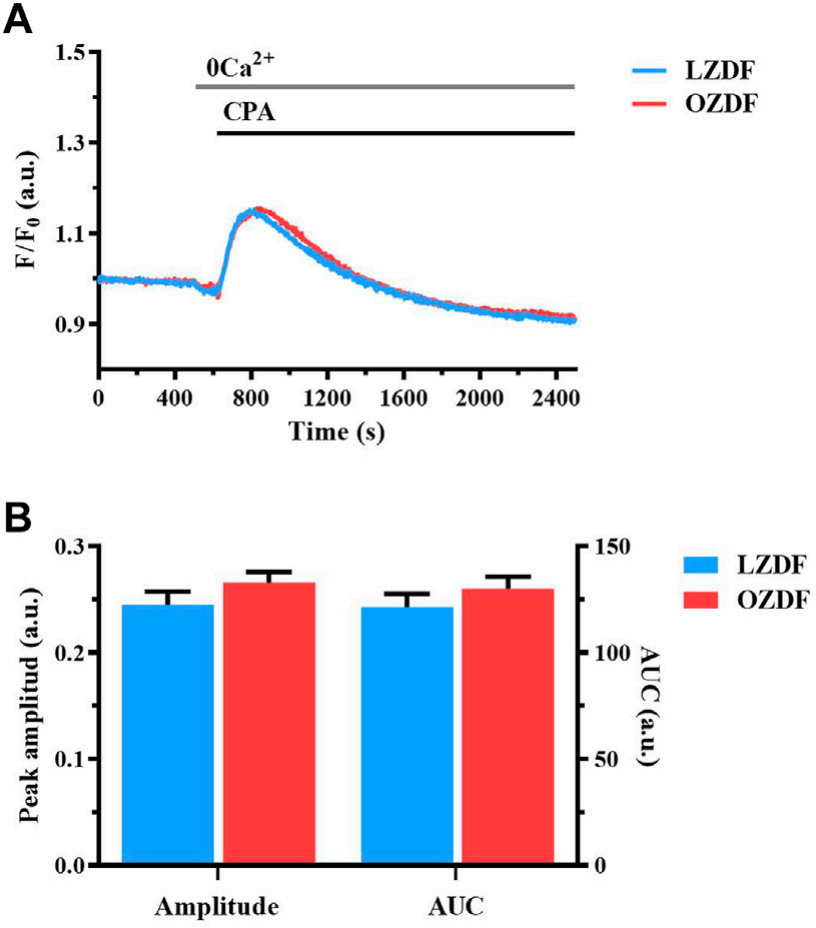
T2DM does not alter SR Ca^2+^ content in rat aortic VSMCs. Mean traces of the Ca^2+^ signal evocked by SERCA inhibitor, CPA in the absence of extracellular Ca^2+^ in VSMCs from LZDF (blue tracing) and OZDF (red tracing) rats **(A)**. Peak amplitude [**(B)**, left)], area under the curve (AUC) [**(B)**, right)] in VSMCs from LZDF (blue bars) and OZDF (red bars) rats. All parameters are expressed as mean ± SE. Statistical comparison between groups was performed using Student’s t-test. * indicates *p* < 0.05 (*n* = 6; 167 cells for LZDF, *n* = 6; 189 cells for OZDF).

The lower ER Ca^2+^ release evoked by the physiological stimulation of IP_3_Rs with ATP could be due to a reduction in the free Ca^2+^ concentration within the ER. Therefore, we evaluated the amount of releasable free Ca^2+^ in the SR by inhibiting SERCA activity with CPA in the absence of extracellular Ca^2+^ (Berra-Romani et al., 2008; Berra-Romani et al., 2019). The application of CPA (10 µM) induced a slow Ca^2+^ transient corresponding to the passive leak of free SR Ca^2+^ into the cytoplasm through leakage channels that remain to be identified (Figure 4A). Subsequently, the intracellular Ca^2+^ concentration returned to the baseline because of Ca^2+^ extrusion across the sarcolemma by PMCA and NCX. CPA is routinely employed to compare SR or endoplasmic reticulum Ca^2+^ content between different cell types (Lodola et al., 2017; Berra-Romani et al., 2019), including VSMCs (Berra-Romani et al., 2008; Liu et al., 2019). No differences were found in the peak Ca^2+^ amplitude and AUC of the Ca^2+^ response to CPA (Figure 4B). These data indicate that there is no detectable difference in the SR free Ca^2+^ concentration between LZDF and OZDF VSMCs. Overall, these findings suggest that IP_3_-induced SR Ca^2+^ release is somehow tempered in rat aortic VSMCs from OZDF rats and that there are no major differences in PMCA and NCX activity under 0Ca^2+^ conditions between the two animal groups (Figures 4A, B).

### 3.6 T2DM alters SERCA activity in rat aortic VSMCs from OZDF rats

In order to assess whether the Ca^2+^-transporting mechanisms clearing the initial Ca^2+^ peak are altered by T2DM, we separately evaluated the contribution of SERCA, PMCA and NCX to ATP-induced increase in [Ca^2+^]_i_ in the presence and absence of extracellular Ca^2+^. In accord, as anticipated above, extracellular Ca^2+^ entry could change the rate of cytosolic Ca^2+^ clearance from the cytosol. Therefore, the Ca^2+^-removal mechanisms at play during the physiological stimulation with ATP could differ depending on whether Ca^2+^ influx is activated or not.

We first evaluated whether and how T2DM alters SERCA activity by measuring the Ca^2+^ response to ATP (300 µM) in the presence of a selective SERCA inhibitor, CPA (10 µM).

#### 3.6.1 SERCA activity changes during the decay of the Ca^2+^ response to ATP in the presence of extracellular Ca^2+^

**FIGURE 5:**
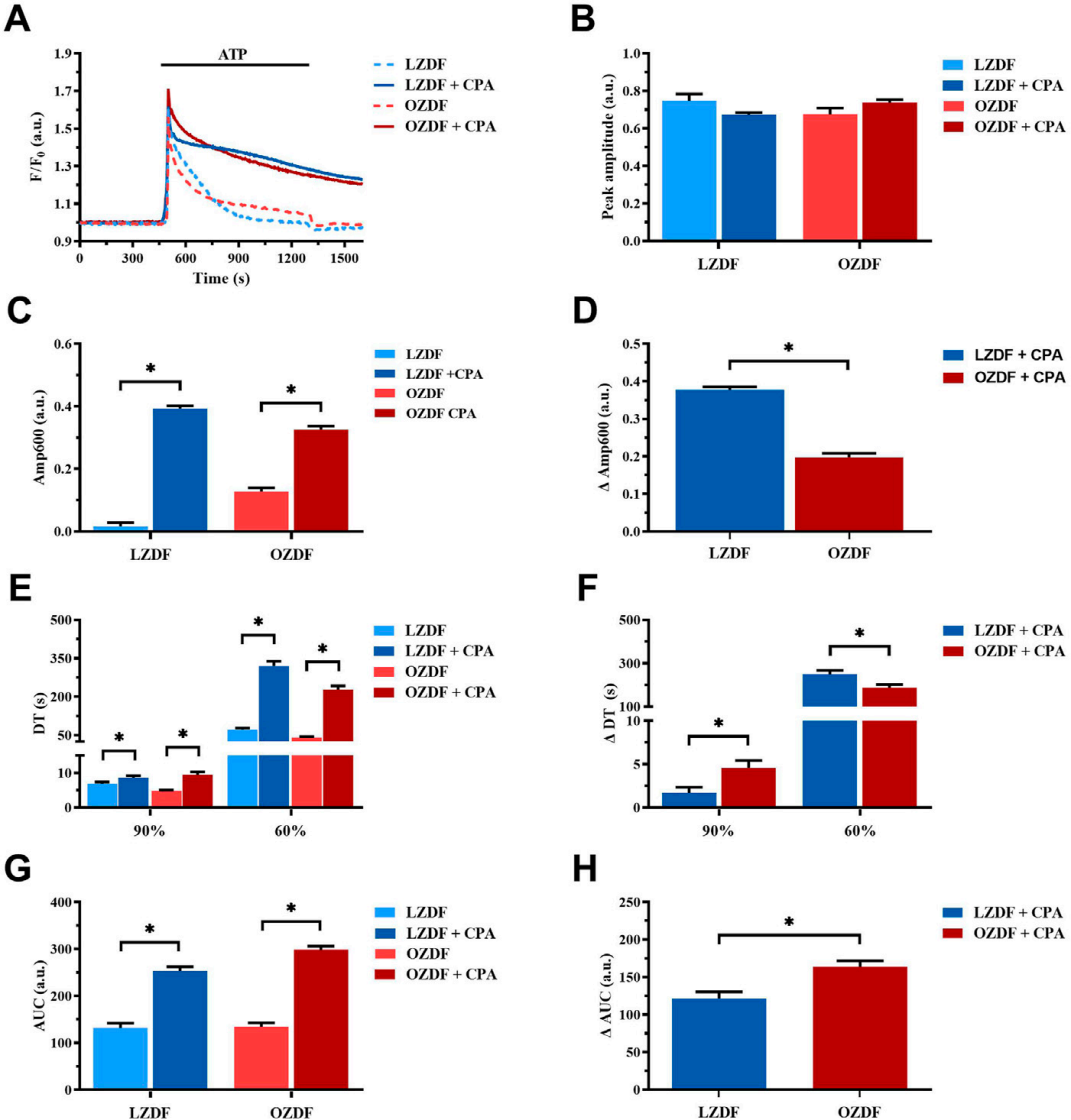
SERCA activity changes during the decay of the Ca^2+^ response to ATP in the presence of extracellular Ca^2+^. Mean traces of the ATP-induced Ca^2+^ transient in the absence (dashed blue line for LZDF and dashed red line for OZDF) and presence (continuous blue line for LZDF and continuous red line for OZDF) of SERCA inhibitor, CPA (10 µM), in VSMCs **(A)**. Peak amplitude **(B)**, amplitude of the late stage of the decay (Amp600) **(C)**, decay time (DT) **(E)** and area under the curve (AUC) **(G)** of the ATP-evoked Ca^2+^ signal in the presence and absence (control) of CPA. Normalized values of Amp600 (ΔAmp600) **(D)**, decay time (ΔDT) **(F)** and AUC (ΔAUC) **(F)** for comparison purposes between LZDF and OZDF groups. All parameters are expressed as mean ± SE. Statistical comparison between groups was performed using Student’s t-test. * indicates *p* < 0.05. *n* = 6; 121 cells for LZDF control, *n* = 6; 127 cells for LZDF + CPA, *n* = 6: 179 cells for OZDF control, *n* = 6; 182 cells for OZDF + CPA.

In aortic VSMCs from LZDF and OZDF rats, the inhibition of SERCA activity with CPA in the presence of extracellular Ca^2+^ did not cause any significant change in the amplitude of the initial Ca^2+^ peak evoked by ATP (300 µM), although there was a trend towards a reduction in LZDF rats and a trend toward an increase in OZDF rats (Figures 5A, B). However, SERCA inhibition with CPA caused a significant (*p* < 0.05) increase in ATP-induced Amp600 in both LZDF and OZDF rats (Figure 5C). Nevertheless, the increase in the ΔAmp600 value induced by CPA was significantly (*p* < 0.05) larger in VSMCs from LZDF as compared to OZDF rats (Figure 5D). Additionally, the pharmacological blockade of SERCA activity induced a significant (*p* < 0.05) increase in the decay time to 90% and 60% in VSMCs from both LZDF and OZDF rats (Figure 5E). However, the increase in the normalized decay time (termed as ΔDT) to both 90% and 60% was significantly (*p* < 0.05) larger in aortic VSMCs from OZDF rats (Figure 5F). In the presence of CPA, the Ca^2+^ response to 300 µM ATP was maintained at such a high plateau level (Figure 5A) that it was not possible to measure the late (30%) clearing rates in both lean and obese animals (Figures 5E,F). Consequently, CPA caused a significant increase (*p* < 0.05) in the AUC value in VSMCs from both LZDF and OZDF rats (Figure 5G), but its elevation (termed as ΔAUC) was significantly (*p* < 0.05) larger in diabetic VSMCs (Figure 5H). These data suggest that SERCA activity is increased in OZDF as compared to LZDF rats during the early stage of decay of the Ca^2+^ transient. However, this effect is dramatically reversed during the late stage of the decay. In accord, in rat aortic VSMCs from OZDF rats stimulated with ATP in the presence of extracellular Ca^2+^, the initial Ca^2+^ peak is sharper due to the faster Ca^2+^ clearance by SERCA, whereas the plateau achieves a larger value because of SOCE upregulation [see Supplementary Figure S5 and (Yan et al., 2020)] and of slower Ca^2+^ clearance by SERCA.

#### 3.6.2 SERCA activity is reduced during the late stage of the decay of the ATP-induced Ca^2+^ transient in the absence of extracellular Ca^2+^

**FIGURE 6:**
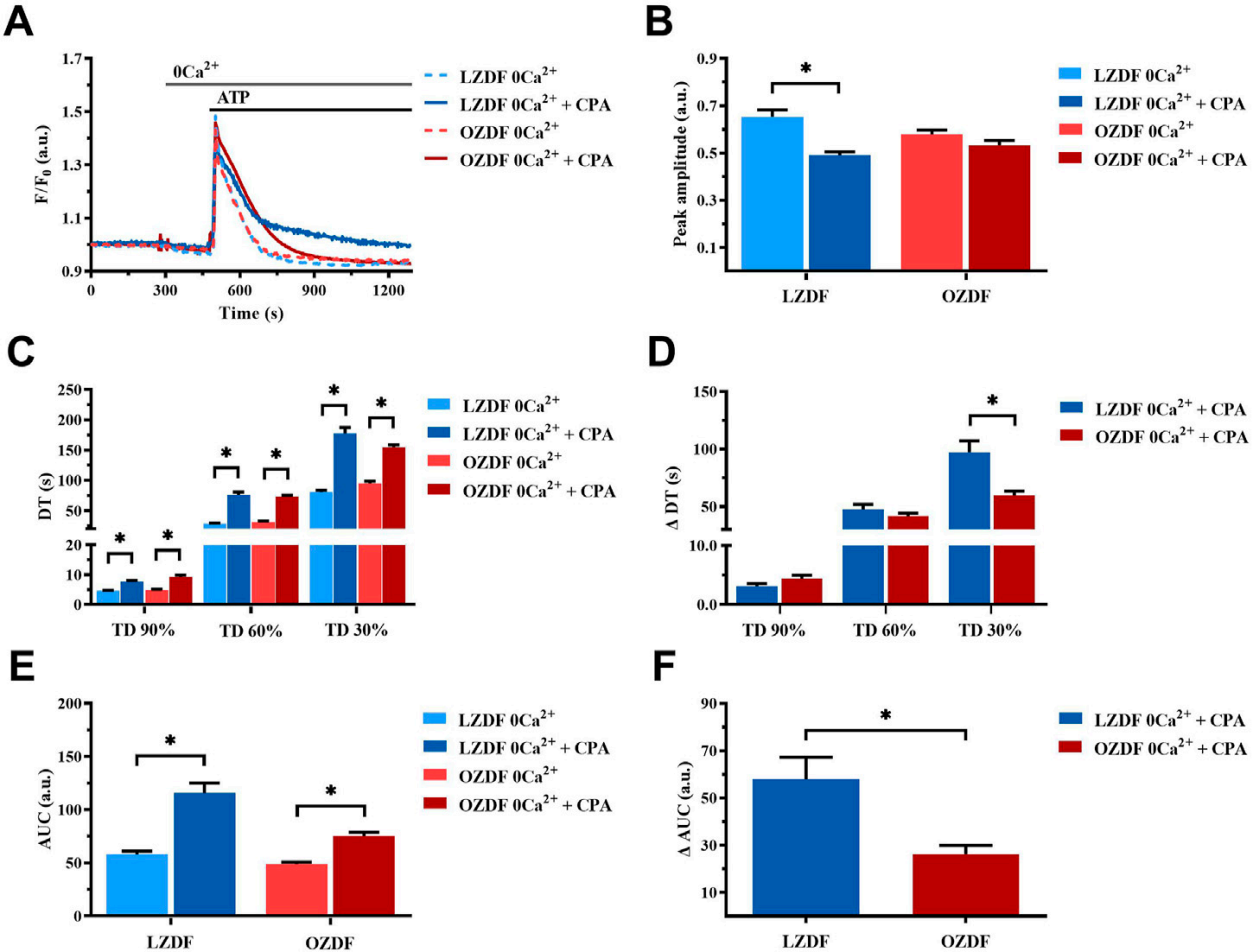
SERCA activity is reduced during the late stage of the decay of the ATP-induced Ca^2+^ transient in 0Ca^2+^. Mean traces of the ATP-induced Ca^2+^ transient in the absence (dashed blue line for LZDF and dashed red line for OZDF) and presence (continuous blue line for LZDF and continuous red line for OZDF) of SERCA inhibitor, CPA (10 µM), in VSMCs **(A)**. Peak amplitude **(B)**, decay time **(C)** and area under the curve (AUC) **(E)** of the ATP-evoked Ca^2+^ signal in the presence and absence (control) of CPA. Normalized values of decay time (ΔDT) **(D)** and AUC (ΔAUC) **(F)** for comparison purposes between LZDF and OZDF groups. All parameters are expressed as mean ± SE. Statistical comparison between groups was performed using Student’s t-test. * indicates *p* < 0.05. *n* = 6; 191 cells for LZDF control, *n* = 7; 242 cells for LZDF CPA 0Ca^2+^, *n* = 6; 204 cells for OZDF control, *n* = 6; 221 cells for OZDF CPA 0Ca^2+^).

In rat aortic VSMCs from LZDF rats, SERCA inhibition with CPA under 0Ca^2+^ conditions significantly (*p* < 0.05) reduced the Ca^2+^ peak amplitude of ATP-induced Ca^2+^ transient as compared to the control Ca^2+^ trace recorded without the inhibitor (Figure 6A, B). In addition, CPA caused a remarkable slowing down of the decay phase, thereby significantly (*p* < 0.05) increasing the decay time to 90%, 60% and 30% and the AUC (Figures 6C,E). As for the OZDF group, SERCA inhibition under 0Ca^2+^ conditions induced a tiny, but not significant decrease in the Ca^2+^ peak amplitude (Figure 6B). However, the decay time to 90%, 60% and 30% and the AUC were again significantly (*p* < 0.05) increased as compared to the control Ca^2+^ trace obtained in the absence of CPA (Figures 6C, E). When the Ca^2+^ response to ATP in both rat groups was normalized, there was no difference in the increase in the decay time (ΔDT) to 90% and 60% (Figure 6D), while the increases in the decay time to 30% (Figure 6D) and in the AUC (ΔAUC; Figure 6F) were significantly (*p* < 0.05) larger in LZDF rats. These findings support the notion that, in rat aortic VSMCs from OZDF rats, SERCA activity is reduced during the late stage of the decay phase (i.e., when the [Ca^2+^]_i_ returns to the 30% of the initial Ca^2+^ peak). In line with this hypothesis, blocking Ca^2+^ clearance with CPA converted the Ca^2+^ transient into a biphasic Ca^2+^ elevation in LZDF but not OZDF rats (Figure 6A). This means that Ca^2+^ pumping by SERCA is reduced during the late stage of the decay phase in diabetic VSMCs and does not significantly contribute to remove cytosolic Ca^2+^. Intriguingly, the larger inhibition of ATP-induced Ca^2+^ release unmasked by 0Ca^2+^ conditions in LZDF VSMCs treated with CPA suggests that SERCA activity can control the rise in [Ca^2+^]_i_ rise in lean but not diabetic animals.

### 3.7 T2DM alters PMCA activity in rat aortic VSMCs

To evaluate PMCA activity in rat aortic VSMCs during T2DM, we used orthovanadate (OV; 500 µM) to inhibit this mechanism during the stimulation with ATP in the presence and absence of extracellular Ca^2+^.

#### 3.7.1 PMCA-dependent Ca^2+^ extrusion is increased during the decay of ATP-induced Ca^2+^ transient in VSMCs from OZDF rats in the presence of extracellular Ca^2+^

**FIGURE 7:**
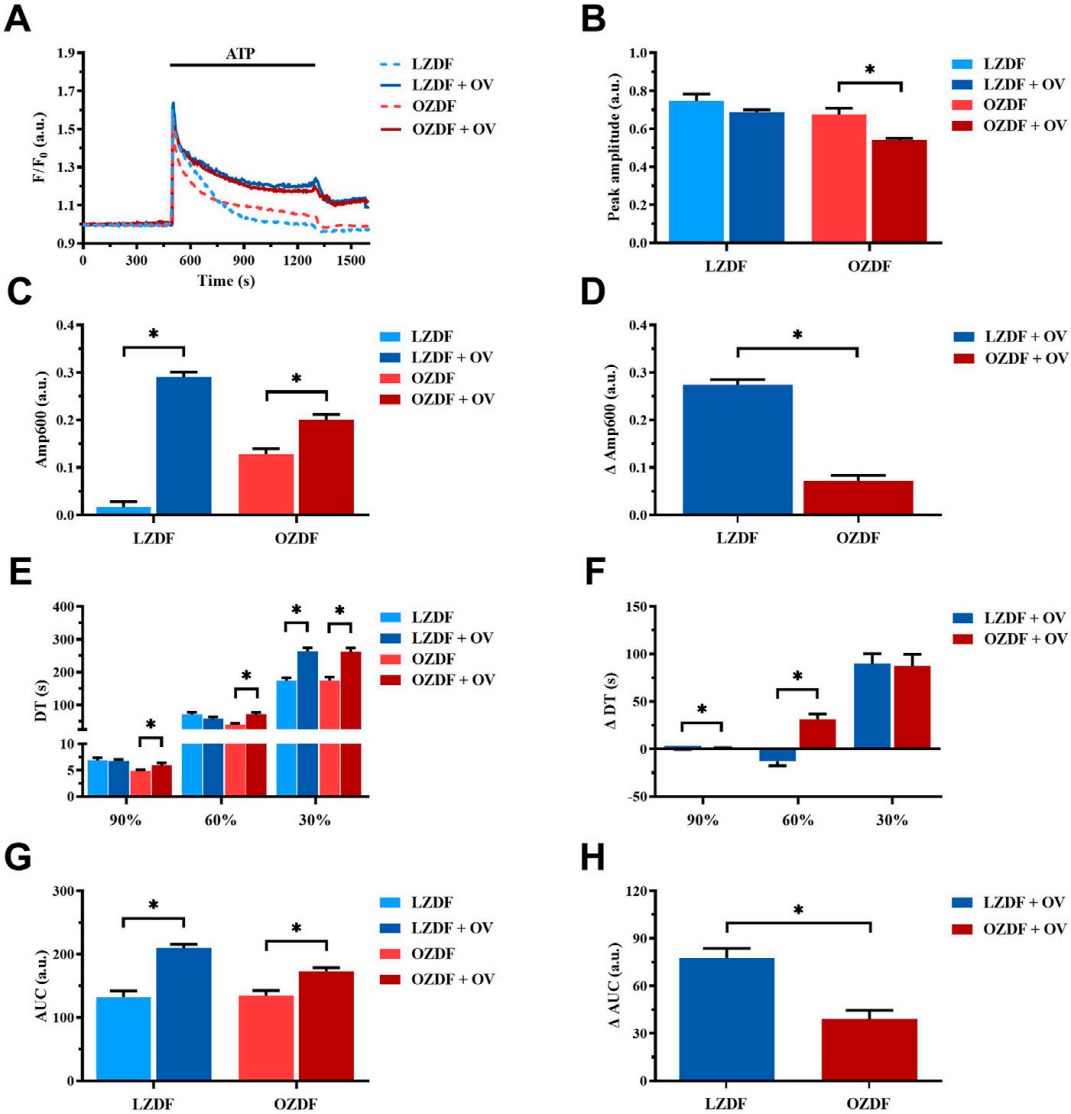
PMCA-dependent Ca^2+^ extrusion is increased during the decay of ATP-induced elevation in [Ca^2+^]_i_ in VSMCs from OZDF rats in the presence of extracellular Ca^2+^. Mean traces of the ATP-induced Ca^2+^ transient in the absence (dashed blue line for LZDF and dashed red line for OZDF) and presence (continuous blue line for LZDF and continuous red line for OZDF) of PMCA inhibitor, sodium orthovanadate (OV, 500 µM) **(A)**. Peak amplitude **(B)**, amplitude of the late stage of the decay (Amp600) **(C)**, decay time **(E)** and area under the curve (AUC) **(G)** of the ATP-evoked Ca^2+^ signal in the presence and absence (control) of OV. Normalized values of Amp600 (ΔAmp600) **(D)**, decay time (ΔDT) **(F)** and AUC (ΔAUC) **(H)** for comparison purposes between LZDF and OZDF groups. All parameters are expressed as mean ± SE. Statistical comparison between groups was performed using Student’s t-test. * indicates *p* < 0.05. (*n* = 6; 121 cells for LZDF control, *n* = 6; 240 cells for LZDF OV, *n* = 6; 179 cells for OZDF control, *n* = 6; 251 cells for OZDF OV.

The inhibition of PMCA activity by using OV in the presence of extracellular Ca^2+^ (Figure 7A) did not affect either the Ca^2+^ peak amplitude (Figure 7B) or the decay time to 90% and 60% of the Ca^2+^ response to ATP in VSMCs from LZDF rats (Figure 7E). Conversely, the value of Amp600 (Figure 7C), the decay time to 30% (Figure 7E) and the AUC (Figure 7G) were significantly (*p* < 0.05) increased in the presence of OV. As to the OZDF group, the inhibition of PMCA activity with OV caused a significant (*p* < 0.05) reduction in the Ca^2+^ peak amplitude (Figure 7B), whereas all the remaining parameters (i.e., Amp600, decay time and AUC) were significantly (*p* < 0.05) increased (Figure 7, panels C, E and G). When the Ca^2+^ response to ATP in both groups of rats was normalized, we found that the increase in the Amp600 value (Figure 7D; ΔAmp600) and in the AUC (ΔAUC; Figure 7H) caused by PMCA inhibition were significantly (*p* < 0.05) larger in VSMCs from LZDF rats as compared to the OZDF group. However, the increase in the decay time to 90% and 60% (ΔDT) was significantly (*p* < 0.05) larger in OZDF rats (Figure 7F). Altogether, these findings suggest that, in the presence of extracellular Ca^2+^, PMCA plays a major role in clearing cytosolic Ca^2+^ at the beginning of the Ca^2+^ response to ATP, while its contribution decreases during the plateau phase, in VSMCs from OZDF rats. In accord, the increase in ΔDT to 90% and 60% induced by OV indicates that the Ca^2+^ pumping rate of PMCA after the initial elevation in [Ca^2+^]_i_ is faster in OZDF VSMCs. Conversely, the lower increase in ΔAmp600 suggests that SERCA plays a minor role in removing Ca^2+^ during the plateau phase and, consequently, its inhibition causes a lower elevation in plateau amplitude. In addition, OV-induced inhibition of ATP-induced ER Ca^2+^ release suggests that PMCA contributes to the propagation of the rise in [Ca^2+^]_i_ following agonist stimulation in diabetic VSMCs. This finding is consistent with the higher activity of PMCA at the beginning of the Ca^2+^ signal discussed above.

#### 3.7.2 PMCA-dependent Ca^2+^ extrusion is reduced during the early stage of the decay of the ATP-induced Ca^2+^ transient in VSMCs from OZDF rats in the absence of extracellular Ca^2+^

**FIGURE 8:**
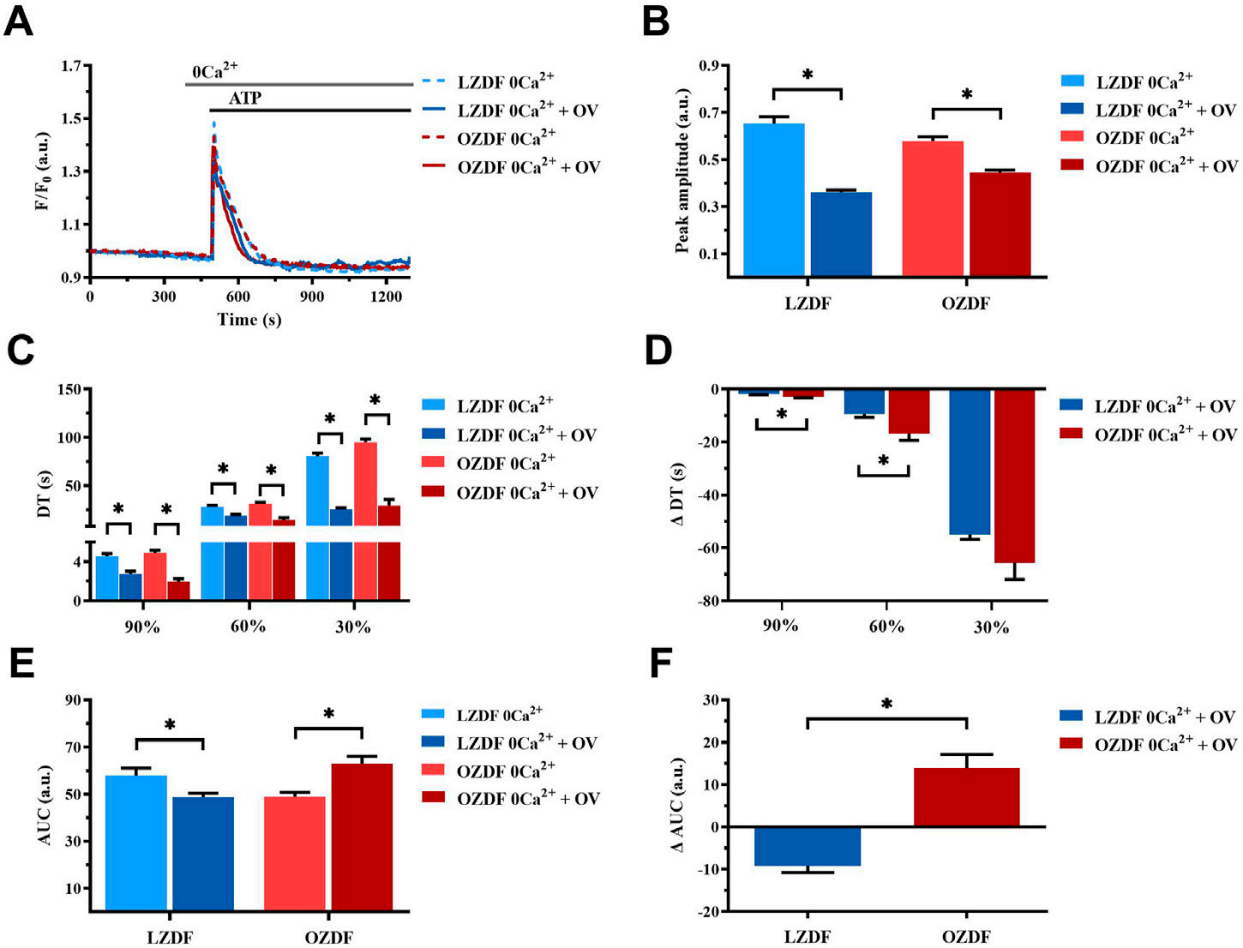
PMCA-dependent Ca^2+^ extrusion is reduced during the early stage of the decay of ATP-induced Ca^2+^ transient in VSMCs from OZDF rats in the absence of extracellular Ca^2+^. Mean traces of the ATP-induced Ca^2+^ transient in the absence (dashed blue line for LZDF and dashed red line for OZDF) and presence (continuous blue line for LZDF and continuous red line for OZDF) of PMCA inhibitor, sodium orthovanadate (OV, 500 µM) in 0Ca^2+^ **(A)**. Peak amplitude **(B)**, decay time **(C)** and area under the curve (AUC) **(E)** of the ATP-evoked Ca^2+^ signal in the presence and absence (control) of OV. Normalized values of decay time (ΔDT) **(D)** and AUC (ΔAUC) **(F)** for comparison purposes between LZDF and OZDF groups. All parameters are expressed as mean ± SE. Statistical comparison between groups was performed using Student’s t-test. * indicates *p* < 0.05. *n* = 6; 191 cells for LZDF control, *n* = 6; 308 cells for LZDF OV 0Ca^2+^, *n* = 6; 204 cells for OZDF control, *n* = 4; 153 cells for OZDF OV 0Ca^2+^.

In rat aortic VSMCs from LZDF rats, the inhibition of PMCA with OV in the absence of extracellular Ca^2+^ (0Ca^2+^) caused a significant (*p* < 0.05) reduction in the Ca^2+^ peak amplitude (Figures 8A, B). Likewise, the decay time to 90%, 60%, and 30% (Figure 8C) and the AUC (Figure 8E) were significantly (*p* < 0.05) reduced. In VSMCs from OZDF rats, pretreatment with OV also caused a significant decrease in the peak amplitude of the Ca^2+^ transient (Figures 8A, B). Similar to LZDF rats, we observed a decrease in the decay time to 90%, 60%, and 30% (Figure 8C), while the AUC was increased in OZDF VSMCs (Figure 8E). When the Ca^2+^ response to ATP in both groups of rats was normalized, the reduction in the decay time to 90% and 60% of the initial Ca^2+^ peak (ΔDT) were significantly larger in OZDF as compared to LZDF rats (Figure 8D). Conversely, the ΔAUC was significantly (*p* < 0.05) larger in the OZDF group compared to LZDF (Figure 8F). These findings confirm that PMCA activity increases during the initial phase of the Ca^2+^ response to ATP in VSMCs from OZDF rats, and that such increase in Ca^2+^ extrusion via PMCA is unmasked by OV.

### 3.8 T2DM alters NCX activity in rat aortic VSMCs

Finally, in order to assess whether and how T2DM alters NCX activity in rat aortic VSMCs, we inhibited this mechanism using the selective inhibitor, SEA0400 (3 µM), in the presence and absence of extracellular Ca^2+^ (0Ca^2+^).

#### 3.8.1 NCX activity changes during Ca^2+^ response evoked by ATP in the presence of extracellular Ca^2+^ in rat aortic VSMCs from OZDF rats

**FIGURE 9:**
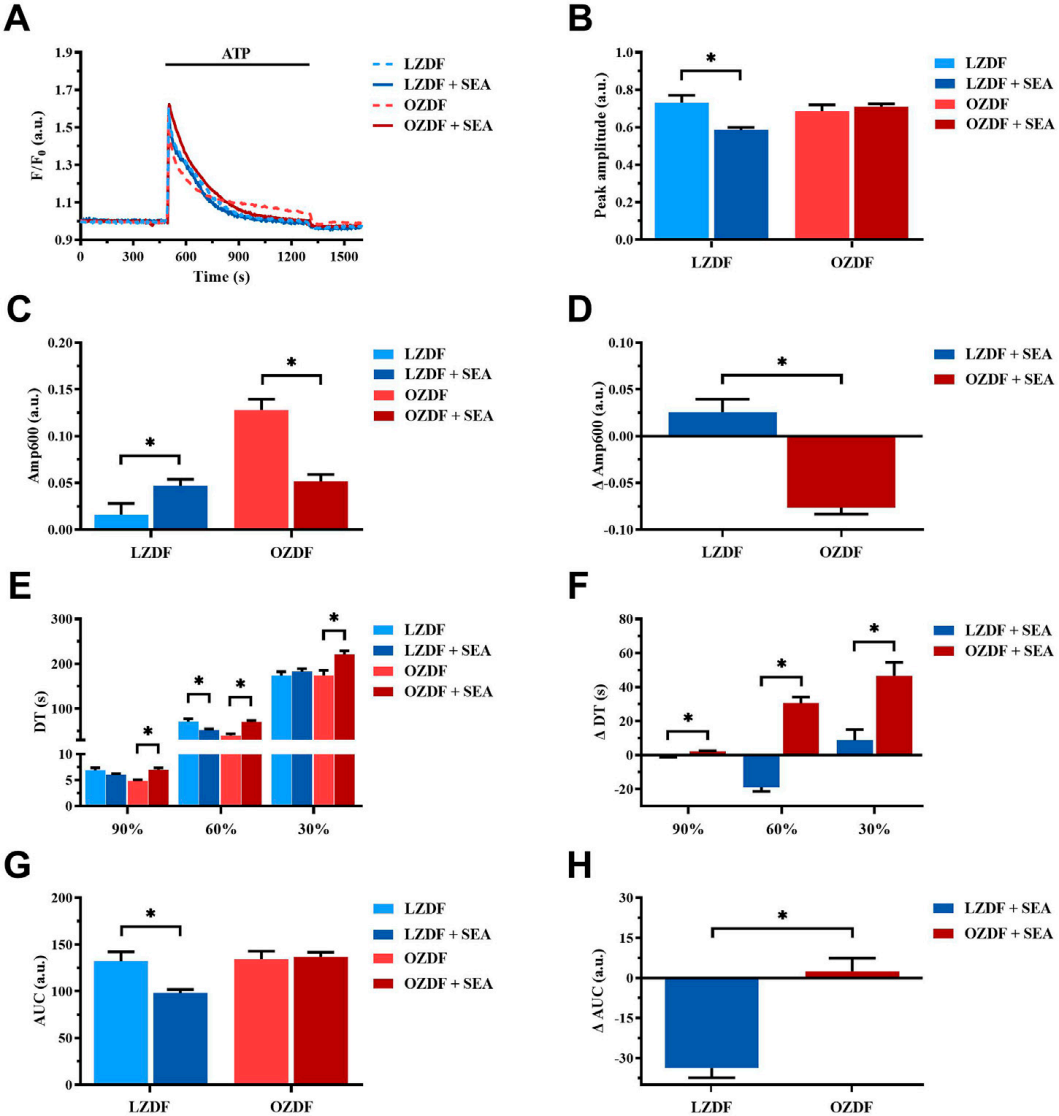
NCX activity changes during the Ca^2+^ response evoked by ATP in the presence of extracellular Ca^2+^ in rat aortic VSMCs from OZDF rats. Mean traces of the ATP-induced Ca^2+^ transient in the absence (dashed blue line for LZDF and dashed red line for OZDF) and presence (continuous blue line for LZDF and continuous red line for OZDF) of NCX inhibitor, SEA0400 (3 µM) **(A)**. Peak amplitude **(B)**, amplitude of the late stage of the decay (Amp600) **(C)**, decay time **(E)** and area under the curve (AUC) **(G)** of the ATP-evoked Ca^2+^ signal in the presence and absence (control) of SEA0400. Normalized values of Amp600 (ΔAmp600) **(D)**, decay time (ΔDT) **(F)** and AUC (ΔAUC) **(H)** for comparison purposes between LZDF and OZDF groups. All parameters are expressed as mean ± SE. Statistical comparison between groups was performed using Student’s t-test. * indicates *p* < 0.05, *n* = 6; 121 cells for LZDF control, *n* = 6; 272 cells for LZDF SEA0400, *n* = 6; 179 cells for OZDF control, *n* = 6; 290 cells for OZDF SEA0400.

In VSMCs from LZDF rats (Figure 9A), blockade of NCX with SEA0400 significantly (*p* < 0.05) reduced the Ca^2+^ peak amplitude (Figure 9B) and increased the Amp600 (Figure 9C) of the Ca^2+^ response to ATP. There were no differences in decay time to 90% and 30% of the total amplitude (Figure 9E), whereas the decay time to 60% (Figure 9E), as well as the AUC (Figure 9G), were significantly (*p* < 0.05) reduced. In OZDF VSMCs (Figure 9A), SEA0400 did not significantly (*p* < 0.05) affect the Ca^2+^ peak amplitude (Figure 9B), although it significantly (*p* < 0.05) reduced the Amp600 (Figure 9C). Conversely, the decay time to 90%, 60%, and 30% were significantly (*p* < 0.05) increased (Figure 9E), while the AUC remained unaltered (Figure 9G). Clearly, there was a significant (*p* < 0.05) difference in the ΔAmp600 (Figure 9D), ΔDT (Figure 9F) ΔAUC between OZDF and LZDF rats (Figure 9H). These findings suggest that NCX modulates the amplitude of the initial Ca^2+^ peak and contributes to extrude cytosolic Ca^2+^ during the plateau phase, but not the early decay phase of the Ca^2+^ response to ATP in LZDF rats. Conversely, NCX is crucial to remove cytosolic Ca^2+^ by acting in the forward mode during all the decay of the initial rise in [Ca^2+^]_i_ but supports Ca^2+^ entry by switching into the reverse mode during the plateau phase, in OZDF rats.

#### 3.8.2 NCX activity is reduced during the early stage of the decay of ATP-induced Ca^2+^ transient in rat aortic VSMCs from OZDF rats in the absence of extracellular Ca^2+^

**FIGURE 10:**
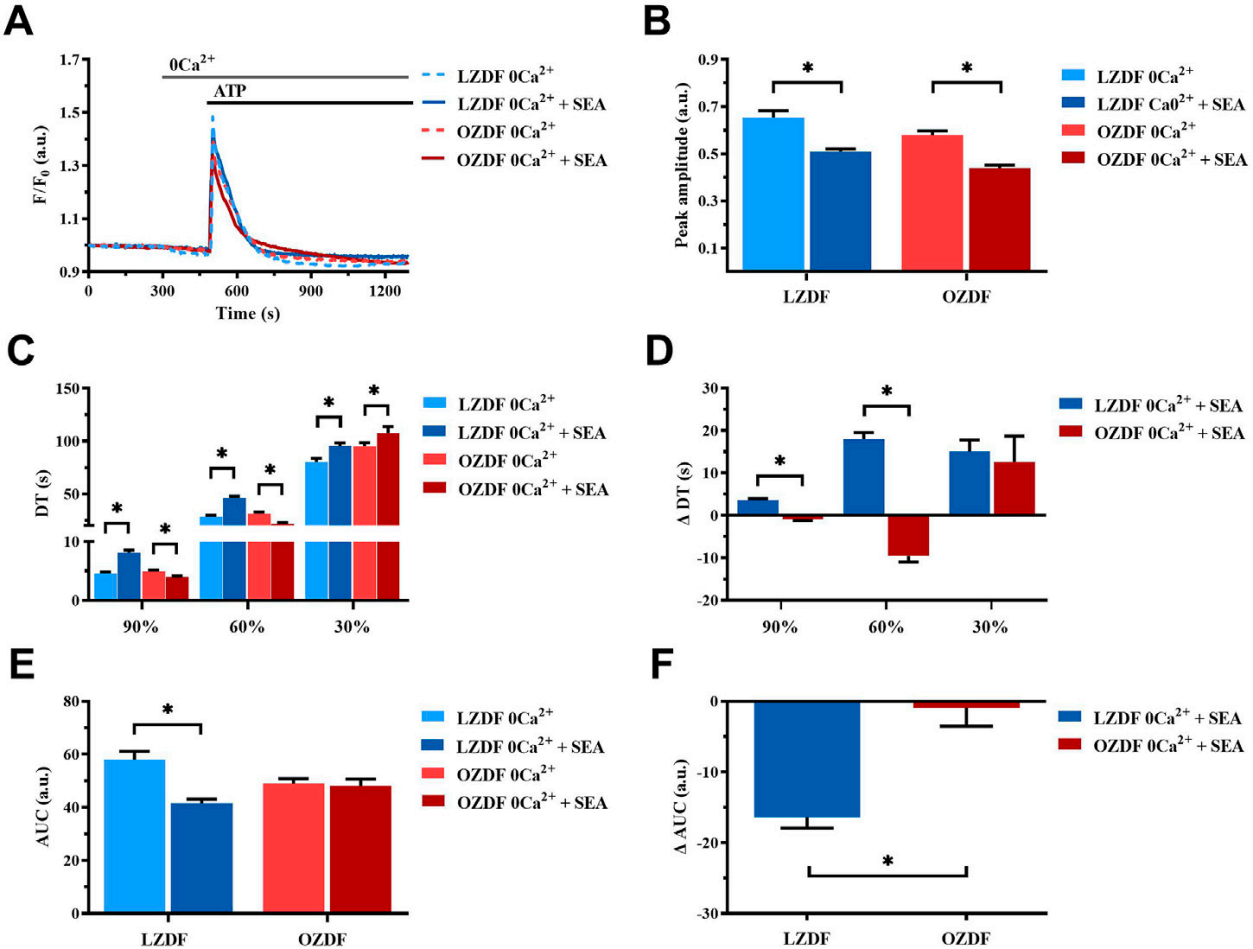
NCX activity is reduced during the early stage of the decay of ATP-induced Ca^2+^ transient in rat aortic VSMCs from OZDF rats in the absence of extracellular Ca^2+^. Mean traces of the ATP-induced Ca^2+^ transient in the absence (dashed blue line for LZDF and dashed red line for OZDF) and presence (continuous blue line for LZDF and continuous red line for OZDF) of NCX inhibitor, SEA0400 (3 µM) in 0Ca^2+^ **(A)**. Peak amplitude **(B)**, decay time **(C)** and area under the curve (AUC) **(E)** of the ATP-evoked Ca^2+^ signal in the presence and absence (control) of SEA0400. Normalized values decay time (ΔDT) **(D)** and AUC (ΔAUC) **(F)** for comparison purposes between LZDF and OZDF groups. All parameters are expressed as mean ± SE. Statistical comparison between groups was performed using Student’s t-test. * indicates *p* < 0.05. *n* = 6; 121 cells for LZDF control, *n* = 6; 199 cells for LZDF SEA0400 0Ca^2+^, *n* = 6; 179 cells for OZDF control, *n* = 6; 162 cells for OZDF SEA0400 0Ca^2+^.

Under 0Ca^2+^ conditions (Figure 10A), the inhibition of NCX with SEA0400 induced a decrease in the Ca^2+^ peak amplitude (Figure 10B) and in the AUC (Figure 10E) of the ATP-evoked Ca^2+^ transient in rat aortic VSMCs from LZDF rats. Conversely, all the decay time were significantly increased as compared to their control values measured in the absence of the inhibitor (Figure 10C). Likewise, in VSMCs from OZDF rats, NCX inhibition under 0Ca^2+^ conditions caused a decrease in the Ca^2+^ peak amplitude of the ATP-induced Ca^2+^ response (Figure 10B) and in the decay time to 90% and 60% (Figure 10C). The decay time to 30% (Figure 10C) increased, while the AUC (Figure 10E) remained unchanged under these conditions. Because of these changes the increase in ΔDT to 90% and 60% was significantly (*p* < 0.05) larger in LZDF VSMCs (Figure 10D), as well as there was a significant (*p* < 0.05) difference in the ΔAUC between OZDF and LZDF rats (Figure 10F). These findings reveal that, under 0Ca^2+^ conditions, NCX modulates the amplitude of the initial Ca^2+^ peak in both LZDF and OZDF rats. Conversely, NCX supports the decline of the Ca^2+^ response to the baseline in LZDF, but not in OZDF VSMCs.

Simultaneous inhibition of SERCA, PMCA and NCX differently alters the Ca^2+^ response to ATP in OZDF as compared to LZDF rat aortic VSMCs.

Finally, we assessed whether and how the simultaneous inhibition of SERCA, PMCA and NCX affects the Ca^2+^ response to ATP both in the presence and absence of extracellular Ca^2+^.

#### 3.8.3 Simultaneous inhibition of SERCA, PMCA and NCX in the presence of extracellular Ca^2+^

**FIGURE 11:**
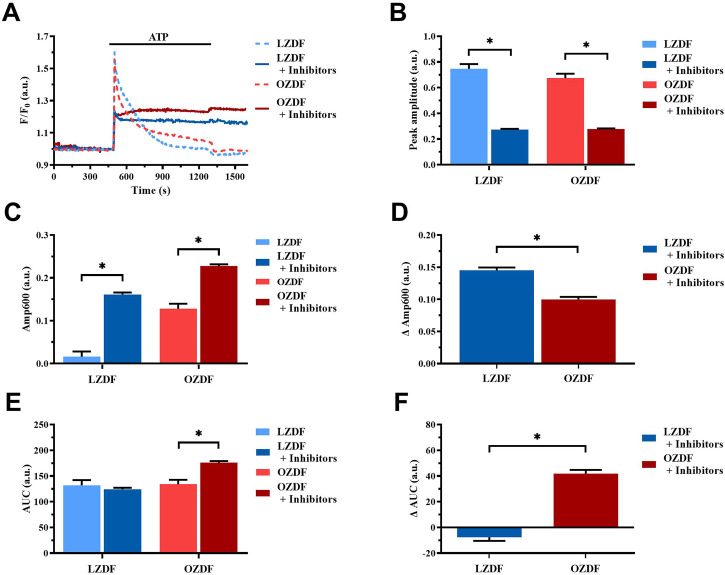
Simultaneous inhibition of SERCA, PMCA and NCX in the presence of extracellular Ca^2+^. Mean traces of ATP-induced Ca^2+^ signals in the absence (dashed blue line for LZDF and dashed red line for OZDF) and presence (continuous blue line for LZDF and continuous red line for OZDF) of CPA (10 µM) + OV (500 µM) + SEA0400 (3 µM) (+inhibitors) in normal extracellular Ca^2+^ concentration **(A)**. Peak amplitude **(B)**, amplitude of the late stage of the decay (Amp600) **(C)** and area under the curve (AUC) **(E)** of the ATP-evoked Ca^2+^ signal in the presence (+inhibitors) and absence (control) of CPA + OV + SEA0400. Normalized values decay time (ΔDT) **(D)** and AUC (ΔAUC) **(F)** for comparison purposes between LZDF and OZDF groups. All parameters are expressed as mean ± SE. Statistical comparison between groups was performed using Student’s t-test. * indicates *p* < 0.05. *n* = 6; 121 cells for LZDF control, *n* = 4; 245 cells for LZDF + Inhibitors, *n* = 6; 179 cells for OZDF control, *n* = 5; 360 cells for OZDF + Inhibitors.

In the presence of extracellular Ca^2+^ (Figure 11A), the Ca^2+^ response elicited by ATP (300 µM) in the presence of CPA (10 µM) + OV (500 µM) + SEA0400 (3 µM) presented a significant (*p* < 0.05) decrease in peak amplitude (Figure 11B) and in Amp600 (Figure 11C) in VSMCs from both LZDF and OZDF rats. These data are consistent with the inhibitory effect exerted by OV (Figure 7B) and SEA0400 (Figure 9B) on peak Ca^2+^ amplitude in aortic VSMCs from, respectively, OZDF and LZDF rats. Likewise, these data are consistent with the evidence that blocking either SERCA (Figure 5C) or PMCA (Figure 7C) activity prevents the recovery of the initial increase in [Ca^2+^]_i_ and enhances the amplitude of the subsequent plateau phase in both animal groups, while blocking NCX activity enhances Amp600 only in LZDF VSMCs (Figure 9C). However, the ΔAmp600 recorded in OZDF VSMCs under these experimental conditions is significantly (*p* < 0.05) lower than in LZDF VSMCs (Figure 11D), which is consistent with a reduction in SERCA and PMCA activity by T2DM. Because of this complex remodelling of the Ca^2+^ handling machinery in obese diabetic rats, the AUC of the Ca^2+^ response undergoes a significant (*p* < 0.05) increase only in VSMCs from OZDF rats (Figures 11E, F).

#### 3.8.4 Simultaneous inhibition of SERCA, PMCA and NCX in the absence of extracellular Ca^2+^

**FIGURE 12:**
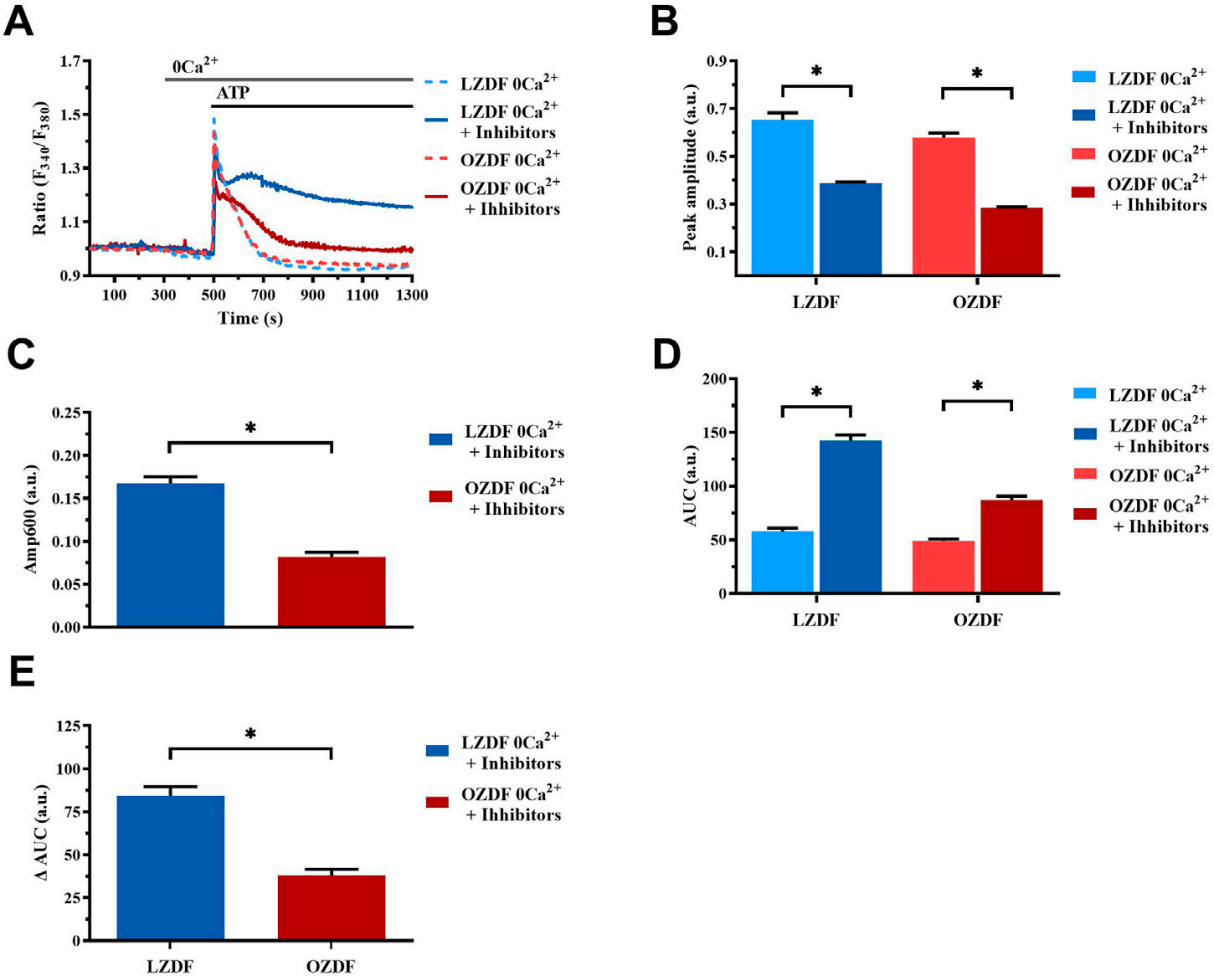
Simultaneous inhibition of SERCA, PMCA and NCX in the absence of extracellular Ca^2+^. Mean traces of the ATP-induced Ca^2+^ signals in the absence (dashed blue line for LZDF and dashed red line for OZDF) and presence (continuous blue line for LZDF and continuous red line for OZDF) of CPA (10 µM) + OV (500 µM) + SEA0400 (3 µM) (+inhibitors) in absence of extracellular Ca^2+^ (0Ca^2+^) **(A)**. Peak amplitude **(B)**, amplitude of the late stage of the decay (Amp600) **(C)** and area under the curve (AUC) **(D)** of the ATP-evoked Ca^2+^ signal in the presence (+inhibitors) and absence (control) of CPA + OV + SEA0400. Normalized values of AUC (ΔAUC) **(E)** for comparison purposes between LZDF and OZDF groups. All parameters are expressed as mean ± SE. Statistical comparison between groups was performed using Student’s t-test. * indicates *p* < 0.05. *n* = 6; 121 cells for LZDF control, *n* = 7; 426 cells for LZDF + Inhibitors, *n* = 6; 179 cells for OZDF control, *n* = 6; 410 cells for OZDF + Inhibitors.

In the absence of extracellular Ca^2+^ (0Ca^2+^) (Figure 12A), the Ca^2+^ response elicited by ATP (300 µM) in the presence of CPA (10 µM) + OV (500 µM) + SEA0400 (3 µM) still presented a significant (*p* < 0.05) decrease in peak amplitude (Figure 12B) in both groups of animals. As expected, under these experimental conditions, the initial increase in [Ca^2+^]_i_ failed to fully recover to the baseline (Figure 12A), thereby resulting in a plateau-like signal that did not return to pre-stimulation levels. Intriguingly, the ΔAmp600 recorded in OZDF VSMCs upon simultaneous blockade of SERCA, PMCA and NCX was significantly (*p* < 0.05) lower than in LZDF VSMCs (Figure 12C). This finding is consistent with the reduction in the activity of all these Ca^2+^-transporting systems under 0Ca^2+^ conditions. Because of this complex remodelling of the Ca^2+^ handling machinery in obese diabetic rats, the AUC of the Ca^2+^ response undergoes a significant (*p* < 0.05) increase only in VSMCs from LZDF rats (Figures 12D, E).

## 4 Discussion

### 4.1 Animal model

As shown in Table 1, at the age of 12 weeks, all the morphological parameters measured (body weight, length, abdominal circumference and epididymal fat) were significantly increased in the OZDF rats in comparison to the LZDF group. The increment in weight concurs with the data previously reported by other research groups (Fridlyand and Philipson, 2006; Tirmenstein et al., 2015; Berra-Romani et al., 2019). Since this could be attributed to an increase in the length of the rat, we calculated the BMI to rule out the possibility that differences in length would interfere with our results. BMI was statistically higher in OZDF rats compared to the LZDF group, evidencing that weight gain is indeed due to obesity, as also reported by other studies (Shiota and Printz, 2012; Wang et al., 2013; Al-Awar et al., 2016; King and Bowe, 2016). The presence of obesity in OZDF rats was also demonstrated by a 405% increase in the amount of adipose tissue formed around the epididymis (epididymal fat). These results confirm that OZDF rats present obesity at the age of 12–16 weeks. Confirming that the animals used for the experimental protocols did indeed develop T2DM, plasma glucose levels 2 hours after intraperitoneally glucose administration (2 g/kg of weight) were more elevated in OZDF rats. This information indicates that OZDF rats are glucose intolerant, which constitutes one of the criteria that according to the ADA, are sufficient to diagnose T2DM in humans (ElSayed et al., 2023).

### 4.2 T2DM shortens the early phase of the decay and increases the plateau amplitude of ATP-induced Ca^2+^ signals in rat aortic VSMCs

The experiments carried out in this study demonstrate that Ca^2+^ handling is altered in VSMCs from OZDF rats. In particular, T2DM causes two important effects on the ATP-evoked biphasic elevation in [Ca^2+^]_i_: 1) a significant reduction in decay time to 90% and 60% of the initial Ca^2+^ peak amplitude (early stage of the decay of [Ca^2+^]_i_ to resting levels) and 2) a significant increase in the amplitude of the plateau (late stage of the decay of [Ca^2+^]_i_ and SOCE activation). Shortening of the early phase of decay could be due to alterations in the activity of the Ca^2+^-clearing mechanisms, such as SERCA, PMCA and NCX.

The role of SERCA (Zhang et al., 2018; Pereira et al., 2022); PMCA (Pande et al., 2006; Baryshnikov et al., 2009) and NCX (Liu et al., 2016; Yang et al., 2020) in clearing cytosolic Ca^2+^ upon agonist stimulation in aortic VSMCs has extensively been reported. However, few studies are available above the effect of DM on the activity of these Ca^2+^-clearing mechanisms VSMCs. In A7r5 cells, hyperglycemia was reported to induce a reduction in SERCA expression and activity (Searls et al., 2010; Tong et al., 2010) and an increase in PMCA and NCX activity (Han et al., 2022). However, our data showing that the decay phase of the initial increase in [Ca^2+^]_i_ evoked by ATP is accelerated in OZDF rat aortic VSMCs suggests that T2DM increases the activity of the Ca^2+^-clearing machinery. As to the increase in plateau amplitude observed in OZDF rat aortic VSMCs, this could clearly be due to SOCE upregulation, as recently shown in (Ma et al., 2020; Zhu et al., 2021). A recent report confirmed that SOCE was enhanced in aortic VSMCs deriving from Zucker diabetic fatty rats due to upregulation of Orai1 protein (Yang et al., 2020). In accord, the Mn^2+^-quenching assay confirmed that extracellular Ca^2+^ entry evoked by the pharmacological depletion of the ER Ca^2+^ store with CPA was remarkably larger in OZDF VSMCs as compared to LZDF. However, the increase in plateau amplitude could also reflect a reduction in the activity of Ca^2+^-extruding mechanisms and/or an increase in Ca^2+^ entry through the reverse mode of NCX (Zhang et al., 2005; Pulina et al., 2010; Liu et al., 2016; Berra-Romani et al., 2019).

### 4.3 T2DM reduces ATP-induced SR Ca^2+^ release in rat aortic VSMCs

In order to elucidate the mechanism(s) whereby T2DM alters the Ca^2+^ response to ATP in rat aortic VSMCs, we first stimulated the cells under 0Ca^2+^ conditions. This condition is widely employed to assess the role played by extracellular Ca^2+^ in shaping peak amplitude, decay phase and plateau amplitude of the Ca^2+^ response to extracellular stimulation (Urena et al., 2007; Berra-Romani et al., 2008; Linde et al., 2012). Ca^2+^ removal from the extracellular solution caused a significant decrease in Ca^2+^ peak amplitude and abolished the plateau phase, thus converting the biphasic Ca^2+^ response to ATP in a transient elevation in [Ca^2+^]_i_ in VSMCs from both animal groups. However, ATP-induced SR Ca^2+^ release was remarkably lower in rat aortic VSMCs from OZDF rats. Enhanced SOCE could therefore shift the amplitude of the initial Ca^2+^ peak evoked by ATP in OZDF VSMCs to the same amplitude as that induced in LZDF cells, which present a larger IP_3_-induced SR Ca^2+^ release but lower SOCE. The reduced Ca^2+^ peak observed under 0Ca^2+^ conditions can be attributed to multiple factors, including downregulation of P2Y receptors, reduced phospholipase C (PLC) activation, lower IP_3_R and RyR expression and/or activity, and a decrease in SR Ca^2+^ content. However, CPA-induced intracellular Ca^2+^ release, which is a widely employed readout of SR Ca^2+^ content, was not altered in OZDF VSMCs, as also reported in VSMCs from diabetic mice (Velmurugan and White, 2012). In contrast, ATP-evoked intracellular Ca^2+^ release, which is mainly dependent on IP_3_Rs (see below), was significantly reduced in OZDF rat aortic VSMCs. This finding suggests that either P2Y receptors are downregulated or their downstream signalling pathways, such as those culminating in PLC engagement and IP_3_R activation, are compromised by T2DM. A study performed in systemic arterial smooth muscle cells deriving from rat models of T1DM, as well as in VSMCs exposed to high glucose, showed a reduction in IP_3_R expression that attenuated vasopressin-induced Ca^2+^ response (Searls et al., 2010). Conversely, Velmurugan et al. found that, in aortic VSMCs from mice with T2DM, IP_3_R excitability was augmented through the direct interaction with the anti-apoptotic Bcl-2 proteins (Velmurugan and White, 2012). The discrepancy in these results could be due to several factors, such as heterogeneity in the vascular district, animal model, type of DM and progress of the disease. However, the evidence that T2DM increases IP_3_R activity in mouse VSMCs strongly suggests that IP_3_-dependent Ca^2+^ release is also enhanced in rat VSMCs. On the other hand, although DM alters RyR function in contractile VSMCs (Fernandez-Velasco et al., 2014), dedifferentiation induces RyR downregulation in the proliferative phenotype of VSMCs (Berra-Romani et al., 2008; House et al., 2008). Consistently, our Ca^2+^ imaging recordings confirmed that caffeine did not induce Ca^2+^ signals in cultured rat aortic VSMCs. Therefore, RyRs are unlikely to contribute to ATP-induced endogenous Ca^2+^ mobilization in the VSMCs employed in the present investigation. Likewise, ionotropic P2X receptors are lost during the dedifferentiation of VSMCs and cannot contribute to the initial Ca^2+^ peak induced by ATP in the presence of extracellular Ca^2+^ (Erlinge et al., 1998). The evidence discussed below that PMCA inhibition reduces the amplitude of the initial Ca^2+^ peak and that PMCA is engaged by Ca^2+^ entry strongly suggest that PMCA activity is required to promote full IP_3_R activation in T2DM.

### 4.4 T2DM alters SERCA activity in rat aortic VSMCs

The inhibition of SERCA activity with CPA in the presence of extracellular Ca^2+^ elongated the decay phase of the Ca^2+^ transient induced by ATP in both experimental groups. However, this effect was greater during the early phases of decay (i.e., 90% and 60%) in OZDF rats. This observation suggests that Ca^2+^ sequestration by SERCA is enhanced during the initial stage of the Ca^2+^ transient and affects the early phase of decay in OZDF VSMCs. Furthermore, SERCA inhibition by CPA caused a larger increase in Amp600, which is a readout of plateau amplitude, in LZDF as compared to OZDF rats. This finding indicates that SERCA activity could decrease during the later stages of the decay phase, which would result in less removal of Ca^2+^ during the plateau phase. Therefore, the larger Amp600 recorded in OZDV VSMCs could be underlain by the combination of enhanced SOCE and reduced SERCA activity. Both an increase and a decrease in SERCA activity in the presence of DM have been reported. Particularly, in dyslipidemic diabetic pigs, Hill et al. found an increase in Ca^2+^ buffering by SERCA2 in coronary artery VSMCs isolated from dyslipidemic diabetic pigs (Hill et al., 2003). Conversely, in rat aortic VSMCs harvested from two different models of T1DM, Searls et al. found a decrease in the expression of SERCA2 and SERCA3, thereby reducing Ca^2+^ and attenuating the Ca^2+^ response to vasopressin (Searls et al., 2010). Downregulation of SERCA was also observed in cultured rat VSMCs exposed to high glucose, although this did not produce any significant alteration in the Ca^2+^ response to phenylephrine (El-Najjar et al., 2017). Interestingly, the protein expression of SERCA2B, which represents the main SERCA isoform in proliferating VSMCs (Berra-Romani et al., 2008) can be significantly enhanced in the thoracic and abdominal aortas of OZDF rats (Berra-Romani et al., 2019). Conversely, SERCA2B activity can be negatively modulated by oxidative stress (Berra-Romani et al., 2019; Negri et al., 2021), which is dramatically enhanced in OZDF rats (Chinen et al., 2007). It has been recently proposed that an early increase in [Ca^2+^]_ i_ can stimulate the production of reactive oxygen species (ROS) via the Ca^2+^-dependent recruitment of NADPH oxidase 5 (NOX5) (Negri et al., 2021). Additionally, ROS could be generated upon NOX2 activation by Gq-protein Coupled Receptors (Negri et al., 2021), such as purinergic P2Y receptors. Therefore, we hypothesize that the initial increase in [Ca^2+^]_ i_ evoked by ATP is rapidly cleared by SERCA2B in rat aortic VSMCs from OZDF rats due to its increased expression as compared to LZDF animals. However, ATP stimulation could also result in NOX2 and/or NOX5 activation, thereby exacerbating the ongoing oxidative stress and inhibiting SERCA2B activity during the plateau phase. Overall, our findings suggest that SERCA activity is increased during the initial phase of the Ca^2+^ response to an IP_3_-producing autacoid, such as ATP, but the decreases during the later stages of the increase in [Ca^2+^]_i_ in aortic VSMCs from OZDF rats.

### 4.5 T2DM alters PMCA activity in rat aortic VSMCs

The next Ca^2+^-clearing mechanism evaluated in the present investigation was PMCA. In both experimental groups, OV caused a larger increase in Amp600 in LZDF as compared to OZDF rats. This finding indicates that also PMCA activity is reduced during the plateau phase of the Ca^2+^ response to ATP in diabetic animals. It turns out that blocking Ca^2+^ extrusion via PMCA with OV causes a larger accumulation of cytosolic Ca^2+^ in LZDF as compared to OZDF VSMCs. It is, therefore, plausible to conclude that the increase in plateau amplitude observed in diabetic rat aortic VSMCs is also due to a decrease in PMCA activity. As to the early phase of the Ca^2+^ signal, the decay time to 90% and 60% of the initial Ca^2+^ peak were significantly increased in OZDF, but not in LZDF VSMCs. This finding suggests that Ca^2+^ extrusion through PMCA is increased by T2DM at the onset of ATP-evoked increase in [Ca^2+^]_i_. Notably, OV reduced the Ca^2+^ peak amplitude in diabetic but not lean VSMCs. This finding suggests that enhanced PMCA activity is somehow required to propagate the rise in [Ca^2+^]_i_ following agonist stimulation, as described in human platelets (Jones et al., 2010). The increase in PMCA activation could be attributed to enhanced SOCE, which can engage PMCA with high spatio-temporal efficiency and thereby finely tune IP_3_-evoked SR Ca^2+^ release during the rising phase of the Ca^2+^ signal.

Conversely, when PMCA was inhibited in the absence of extracellular Ca^2+^, the decay time at 90%, 60% and 30% were significantly reduced in both animal groups. This finding strongly suggests that PMCA plays a minor role in buffering the initial increase in [Ca^2+^]_i_ under 0Ca^2+^ conditions. Furthermore, the amplitude of the initial Ca^2+^ peak was significantly decreased also in LZDF rat aortic VSMCs, which means that extracellular Ca^2+^ entry can substitute PMCA to regulate IP_3_-dependent SR Ca^2+^ release in lean animals. Interestingly, also CPA reduced the amplitude of the initial Ca^2+^ peak in LZDF VSMCs stimulated with ATP only under 0Ca^2+^ conditions, thereby indicating that SERCA interacts with PMCA to finely tune IP_3_-induced SR Ca^2+^ mobilization in non-diabetic VSMCs in the absence of Ca^2+^ entry.

These results, therefore, set extracellular Ca^2+^ as a regulator of PMCA in rat aortic VSMCs. PMCA activity is likely to be increased during the early phase of Ca^2+^ response evoked by ATP in the presence of extracellular Ca^2+^ in OZDF rat aortic VSMCs, while it declines during the subsequent plateau phase. In contrast, PMCA does not seem to have a relevant role under 0Ca^2+^ conditions in both animal groups. A functional coupling between SOCE and PMCA has long been known. Extracellular Ca^2+^ incoming through Orai1 channels can stimulate adjacent PMCA in a calmodulin-dependent manner and promote Ca^2+^ extrusion across the plasma membrane (Klishin et al., 1998; Snitsarev and Taylor, 1999). PMCA might not be engaged under 0Ca^2+^ conditions, so that IP_3_-released Ca^2+^ is removed by alternative Ca^2+^-clearing mechanisms that present a higher extrusion rate and therefore accelerate the decay phase of the Ca^2+^ transient in both animal groups. The subsequent inhibition of PMCA activity during the late phase of decay even in the presence of extracellular Ca^2+^ could again reflect the enhanced oxidative stress imposed to diabetic VSMCs during the development of the Ca^2+^ signal. In accord, it has been demonstrated that ROS inhibit PMCA activity and thereby favour the accumulation of cytosolic Ca^2+^ (Kim et al., 2018), which is consistent with the increase in plateau amplitude in OZDF VSMCs.

Scarce information is available regarding the mechanisms by which T2DM affects PMCA in VSMCs; however, our results resemble those reported by El-Najjar et al., who showed that PMCA activity in rat aortic VSMCs exposed to high extracellular glucose is enhanced due to the increase in PMCA4 expression (El-Najjar et al., 2017). Similar results were observed in VSMCs from coronary arteries of diabetic and dyslipidemic pigs (Hill et al., 2003). Nevertheless, neither study evaluated whether PMCA activity decreases during the Ca^2+^ response to an IP_3_-producing autacoid.

### 4.6 T2DM alters NCX activity in rat aortic VSMCs

The last Ca^2+^-clearing mechanism evaluated in the present investigation was NCX. As described for SERCA and PMCA, NCX inhibition elicited different effects in lean vs. diabetic rats. In the presence of extracellular Ca^2+^, SEA0400 reduced the amplitude of the initial Ca^2+^ peak in LZDF but not OZDF VSMCs. Therefore, NCX is likely to regulate IP_3_-induced SR Ca^2+^ release in rat aortic VSMCs from lean animals. Furthermore, blocking NCX activity did not remarkably affect the decay phase, but increased Amp600, of the Ca^2+^ response evoked by ATP in LZDF rats. Conversely, SEA0400 slowed down all the decay time and inhibited the plateau phase in OZDF VSMCs. These findings indicate that: 1) NCX operates in the forward (i.e., Ca^2+^ out) mode during the late phase of the Ca^2+^ response to ATP in lean VSMCs and 2) NCX switches from the forward to the reverse-mode (i.e., Ca^2+^ in) during the decay phase of the Ca^2+^ response evoked by ATP in diabetic VSMCs. The Ca^2+^-clearing activity of NCX during the early phase of the ATP-evoked increase in [Ca^2+^]_i_ could contribute to explain its sharper decline in OZDF VSMCs. Conversely, under 0Ca^2+^ conditions, Ca^2+^ extrusion via NCX seems to be decreased in diabetic VSMCs. In accord, blocking NCX activity accelerated the decay rate, which means that NCX is unlikely to clear cytosolic Ca^2+^ in the absence of Ca^2+^ entry. On the other hand, SEA0400 slows down the clearing rate in LZDF VSMCs, which indicates that in lean animals NCX drives the recovery of the Ca^2+^ transient to the baseline only under 0Ca^2+^ conditions.

Five Similar to SERCA, it is still unclear how T2DM affects NCX activity in VSMCs. A study performed by El-najjar et al. showed that, in VSMCs exposed to high glucose, there were no alterations in NCX expression or activity (El-najjar et al., 2017). Conversely, NCX activity was enhanced in cardiomyocytes isolated from db/db obese type 2 diabetic mice (Pereira et al., 2006). Future studies are required to understand whether NCX and PMCA expression are altered in aortic VSMCs from OZDF rats. Conclusion.

Herein, we provided the first demonstration that the Ca^2+^-transporting machinery is deranged in aortic VSMCs from diabetic obese rats. The Ca^2+^ pumping activity of SERCA and PMCA is increased by T2DM during the early phase of the Ca^2+^ transient, whereas it is remarkably slowed down during the following plateau phase. Similarly, NCX contributes to buffer the initial increase in [Ca^2+^]_i_ by operating in the reverse-mode, but it then reverses into the Ca^2+^-entry mode during the plateau phase. Therefore, the larger plateau observed in rat aortic VSMCs from OZDF rats is not only due to SOCE upregulation, as shown by others and confirmed in the present study, but also to the lower Ca^2+^ extrusion via PMCA and SERCA and to the activation of the reverse-mode of NCX. This latter observation suggests that a Na^+^-permeable channel is recruited or enhances its activity in diabetic VSMCs challenged with ATP. The non-selective cation channel, TRP Canonical 6 (TRPC6), can be gated by diacylglycerol produced upon PLC activation and generates local cytosolic Na^+^ transients beneath the plasma membrane that drive NCX-mediated Ca^2+^ entry in purinergically stimulated rat aortic VSMCs (Poburko et al., 2007). Future work will have to assess whether TRPC6 activation is enhanced by T2DM and contributes to further elevate the [Ca^2+^]_i_ during the plateau phase. Preliminary evidence showed that TRPC6 protein was upregulated in caudal artery smooth muscle from Type 2 diabetic Goto-Kakizaki rats (Mita et al., 2010), whereas TRPC3 protein expression was increased in platelets of patients affected by T2DM (Zbidi et al., 2009).

Intriguingly, extracellular Ca^2+^ entry is required to engage PMCA and the forward mode of NCX during the decay phase. The functional coupling between Orai1, i.e., the pore-forming subunit of SOCs, and PMCA has been firmly established, while a straight-forward relationship between SOCE and NCX-mediated Ca^2+^ extrusion is yet to be reported. The faster decay of [Ca^2+^]_i_ observed in diabetic rat aortic VSMCs challenged with ATP in the absence of extracellular Ca^2+^ and upon inhibition of either PMCA or NCX activity suggests the involvement of an alternative Ca^2+^-clearing mechanism. Mitochondrial have been shown to rapidly buffer Ca^2+^ at high [Ca^2+^]_i_ in rat systemic arterial smooth muscle cells (McCarron and Muir, 1999; Kamishima et al., 2000). Although this issue remains highly controversial, the rate of mitochondrial Ca^2+^ uptake could be increased by T2DM (Belosludtsev et al., 2020). Intriguingly, the simultaneous blockade of SERCA, PMCA and NCX activity in the absence of extracellular Ca^2+^ results in a plateau-like elevation in [Ca^2+^]_i_ that presents a higher Amp600 in VSMCs from LZDF rats. This finding implies that an additional Ca^2+^-removal mechanism intervene to buffer, at least partially, the initial rise in[Ca^2+^]_I_ elicited by ATP in rat aortic VSMCs from OZDF rats. These alterations in the Ca^2+^ clearing machine in aortic VSMCs could exacerbate vascular dysfunction in diabetes. For instance, the aberrant increase in [Ca^2+^]_i_ observed could enhance VSMC proliferation and migration, thereby favoring atherosclerotic plaques and vascular calcification. In addition, cytosolic Ca^2+^ overload in VSMCs could exacerbate oxidative stress within the vascular wall (Negri et al., 2021).

## Data Availability

The raw data supporting the conclusion of this article will be made available by the authors, without undue reservation.
