# Supplementary material for: Alterations of the Ca2+ clearing mechanisms by type 2 diabetes in aortic smooth muscle cells of Zucker diabetic fatty rat
Source: Front Physiol. 2023 May 11;14:1200115. doi: 10.3389/fphys.2023.1200115 (PMC10213752; doi:10.3389/fphys.2023.1200115)
Supplement: Supplementary file 1 [file DataSheet2.PDF]

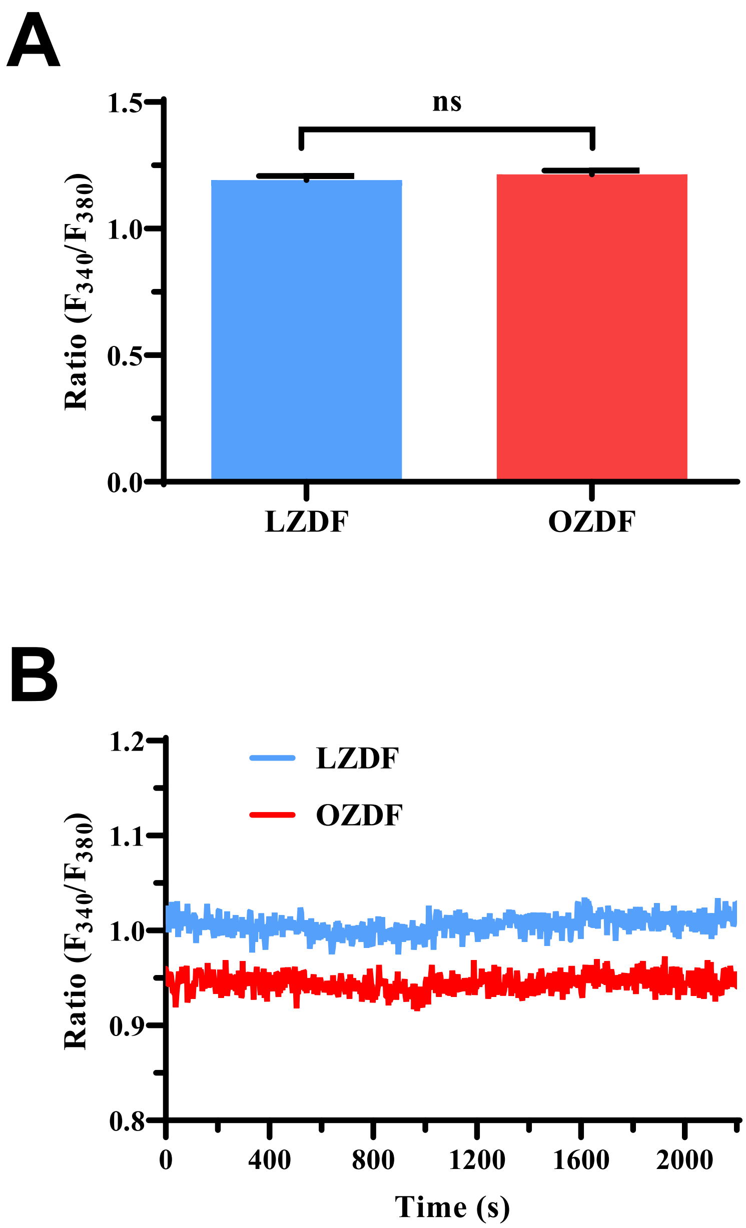

Figure S2. Resting Ca^{2+} levels. (**A**) Mean ± SE of the resting Ca^{2+} levels measured in LZDF (blue bar) and OZDF (red bar). Statistical comparison between groups was performed using Student's t-test. ns (non-significant) indicates $p \geq 0.05$. n=4; 127 cells for LZDF; n=5; 182 cells for OZDF rats. Typical Ratio (F_{340}/F_{380}) recordings obtained from a single aortic VSMC loaded with Fura-2 from LZDF (blue line) and OZDF (red line) rats. (**B**) Long-term recording of Fura-2 fluorescence did not detect any spontaneous increase in $[Ca^{2+}]_i$ in aortic VSMCs from both OZDF and LZDF rats.
