# Supplementary material for: Alterations of the Ca2+ clearing mechanisms by type 2 diabetes in aortic smooth muscle cells of Zucker diabetic fatty rat
Source: Front Physiol. 2023 May 11;14:1200115. doi: 10.3389/fphys.2023.1200115 (PMC10213752; doi:10.3389/fphys.2023.1200115)
Supplement: Supplementary file 2 [file DataSheet4.PDF]

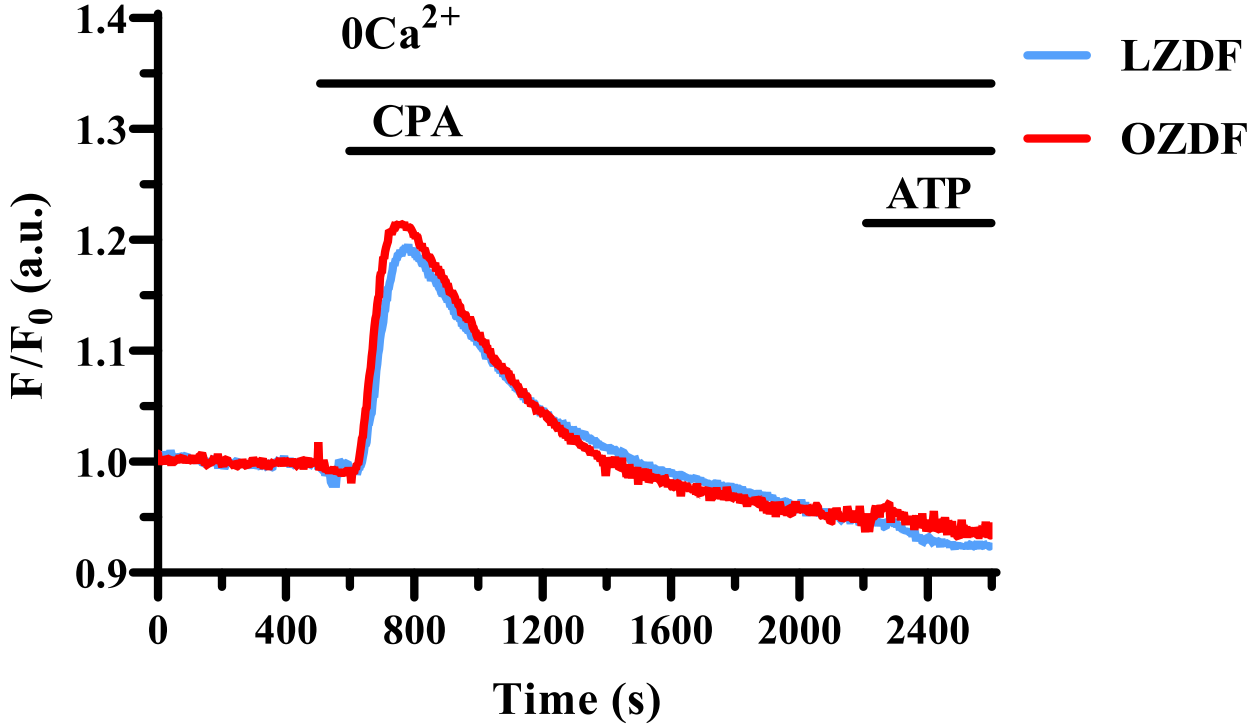

Figure S4. Depletion of the ER Ca^{2+} store with CPA prevented the subsequent Ca^{2+} response to ATP. Depletion of the ER Ca^{2+} store with cyclopiazonic acid (CPA; 10 µM) prevented the subsequent Ca^{2+} response to ATP (300 µM) in aortic VSMC from LZDF (blue line) and (OZDF (red line) rats loaded with Fura-2.
