# Supplementary material for: Alterations of the Ca2+ clearing mechanisms by type 2 diabetes in aortic smooth muscle cells of Zucker diabetic fatty rat
Source: Front Physiol. 2023 May 11;14:1200115. doi: 10.3389/fphys.2023.1200115 (PMC10213752; doi:10.3389/fphys.2023.1200115)
Supplement: Supplementary file 3 [file DataSheet3.PDF]

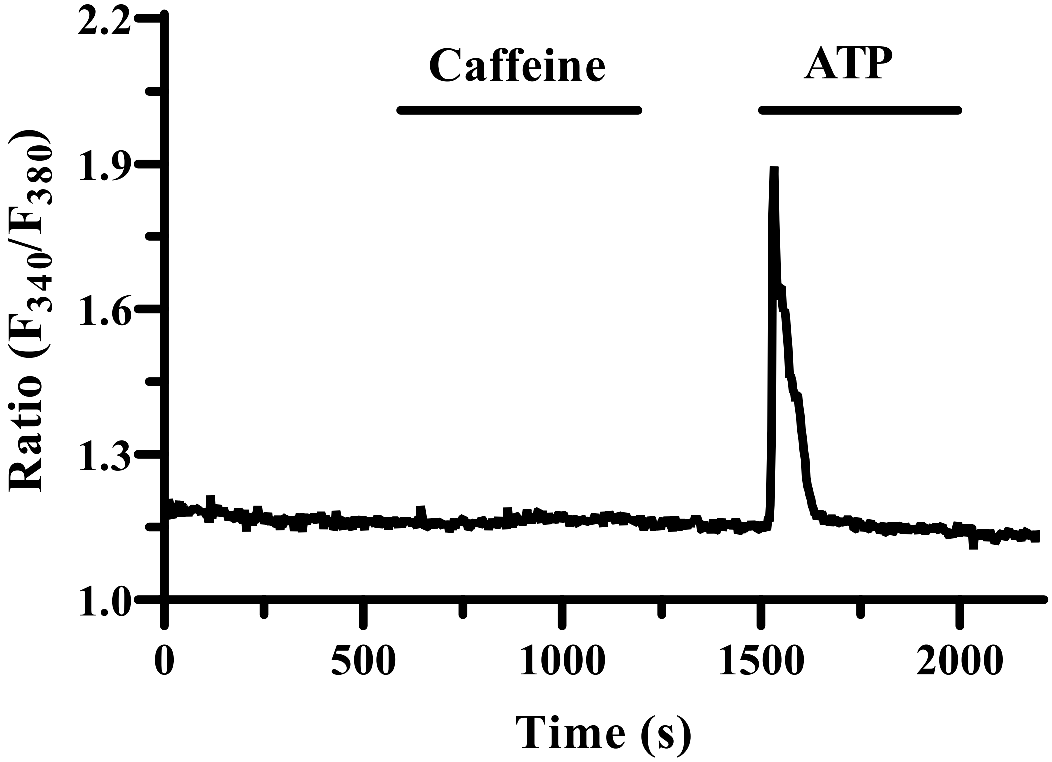

Figure S3. Effect of caffeine on Ca^{2+} signal in aortic VSMCs. Typical Ratio (F_{340}/F_{380}) recordings obtained from a single aortic VSMC loaded with Fura-2 from LZDF rats and stimulated with caffeine (10 mM) and ATP (20 µM).
