# Supplementary material for: Alterations of the Ca2+ clearing mechanisms by type 2 diabetes in aortic smooth muscle cells of Zucker diabetic fatty rat
Source: Front Physiol. 2023 May 11;14:1200115. doi: 10.3389/fphys.2023.1200115 (PMC10213752; doi:10.3389/fphys.2023.1200115)
Supplement: Supplementary file 4 [file DataSheet1.PDF]

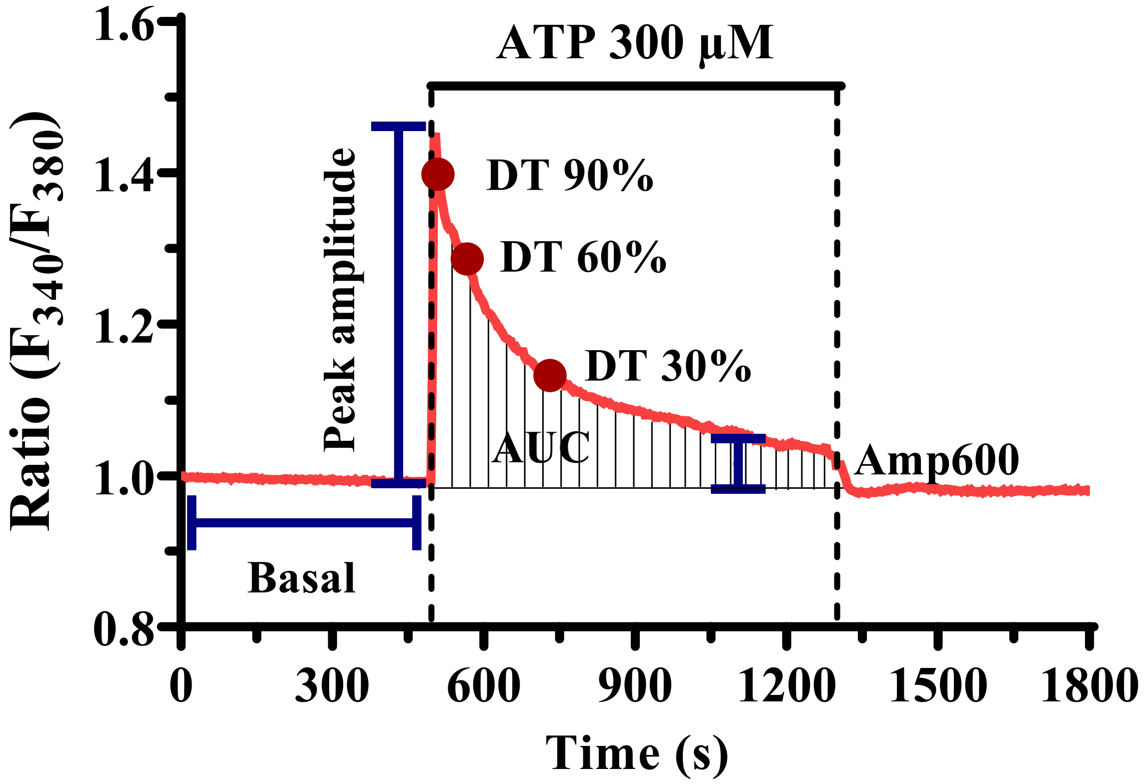


**Figure S1. Data analysis.** The peak amplitude of the $Ca^{2+}$ response was measured as the difference between the Ratio ($F_{340}/F_{380}$) at the peak and the mean Ratio ($F_{340}/F_{380}$) of 500 s baseline (Basal) before the peak. Amplitude of the late stage of the decay (Amp600) was calculated as the difference between the Ratio ($F_{340}/F_{380}$) 600 s after adding the agonist and the mean Ratio ($F_{340}/F_{380}$) of 200 s baseline before the peak of the $Ca^{2+}$ response. The duration of the $Ca^{2+}$ response to ATP was measured as the time it takes the $Ca^{2+}$ signal to be reduced at 90% (DT 90%), 60% (DT 60%) and 30% (DT 30%) of the initial $Ca^{2+}$ peak amplitude (considered as 100%), shorty termed as “decay time” (DT). The area under the curve (AUC) was measured by calculating the integral of each $Ca^{2+}$ tracing from when the ATP is applied until it is removed.
