# Supplementary material for: Alterations of the Ca2+ clearing mechanisms by type 2 diabetes in aortic smooth muscle cells of Zucker diabetic fatty rat
Source: Front Physiol. 2023 May 11;14:1200115. doi: 10.3389/fphys.2023.1200115 (PMC10213752; doi:10.3389/fphys.2023.1200115)
Supplement: Supplementary file 5 [file DataSheet5.PDF]

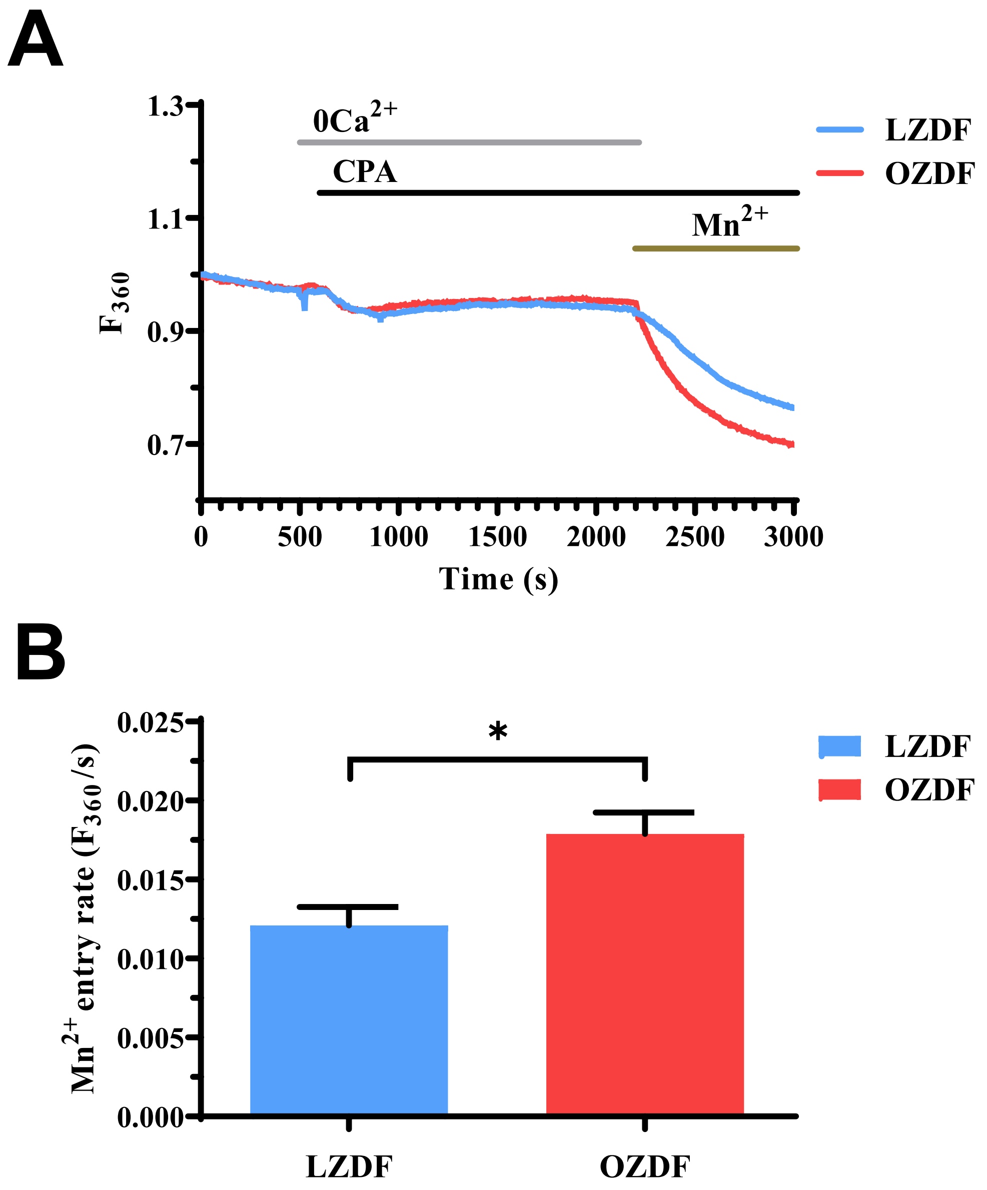


**Figure S5. T2DM enhances $Mn^{2+}$ entry through store-operated channels.** Mean traces of the fluorescence levels during the fura-2-quenching-protocol using SERCA inhibitor, CPA (10 µM), to evaluate $Mn^{2+}$ entry through SOCs in VSMCs from LZDF (blue tracing) and OZDF (red tracing) rats **(A)**. The signal resulting after $MnCl_2$ (100 µM) addition was fitted to a linear regression and $Mn^{2+}$ entry rate was calculated as the slope of it **(B)**. All parameters are expressed as mean ± SE. Statistical comparison between groups was performed using Student's *t*-test. * indicates $p<0.05$. n=6; 115 cells for LZDF, n=6; 129 cells for OZDF).
